# Peritoneal Dialysis -Associated Fibrosis: Emerging Mechanisms and Therapeutic Opportunities

**DOI:** 10.3389/fphar.2025.1635624

**Published:** 2025-08-22

**Authors:** Narayan Prasad, Saurabh Chaturvedi, Harshit Singh, Mary Priyanka Udumula, Atul Rawat, Meyyappan Jeyakumar, Akhilesh Jaiswal, Sachin Kumar, Vikas Agarwal

**Affiliations:** ^1^Department of Nephrology, Sanjay Gandhi Post Graduate Institute of Medical Sciences, Lucknow, Uttar Pradesh, India; ^2^Department of Medical Laboratory Technology and Sciences, School of Allied Health Sciences, Delhi Pharmaceutical Sciences and Research University, New Delhi, India; ^3^ Department of Women’s Health Services, Henry Ford Hospital, Henry Ford Cancer Institute, Detroit, MI, United States; ^4^Department of Nephrology, Sir Ganga Ram Hospital, New Delhi, India; ^5^Department of Clinical Immunology and Rheumatology, Sanjay Gandhi Post Graduate Institute of Medical Sciences, Lucknow, Uttar Pradesh, India

**Keywords:** peritoneal fibrogenesis, epithelial to mesenchymal transition, biomechanical injury in peritoneal membrane, epigenetics and altered gut microbiome, peritoneal dialysis solutions and inflammation, peritonitis and peritoneal angiogenesis, Smad/non-Smad signaling, biomarkers

## Abstract

Peritoneal Dialysis (PD) requires a healthy and functional peritoneal membrane for adequate ultrafiltration and fluid balance, making it a vital treatment for patients with end-stage renal disease (ESRD). The spectrum of PD-associated peritoneal fibrosis encompasses a diverse range of collective mechanisms: peritoneal fibrogenesis, epithelial to mesenchymal transition (EMT), peritonitis, angiogenesis, sub-mesothelial immune cells infiltration, and collagen deposition in the sub-mesothelial compact zone of the membrane that accompany deteriorating membrane function. In this narrative review, we summarize the repertoire of current knowledge about the structure, function, and pathophysiology of the peritoneal membrane, focusing on biomolecular mechanisms and signalling pathways that potentiate the development and progression of peritoneal fibrosis. The article suggests future directions that could enhance our comprehension of the relationship between peritoneal membrane dysfunction and its fibrosis to elucidate the promising targets for therapeutic interventions. A thorough understanding of early events in pathophysiology closely associated with the inflammatory events in peritoneal fibrosis is the logical starting point for identifying new targets rather than concentrating on more downstream effects. Biomarkers are essential for monitoring the progression of peritoneal fibrosis and evaluating the effectiveness of therapeutic interventions. Biomarkers are evolving in concert with new targets and novel agents, and biomarker outcomes offer a means of monitoring the peritoneal membrane’s health. Recent approaches to reducing the etiologies of peritoneal membrane dysfunction, the impact of fibroblast switch, and peritoneal membrane events perturbing fibroblast function are explored and suggest using unique, effective therapeutic strategies to target peritoneal fibrosis and associated complications.

## Key points

• New data supporting the hypothesis that biomechanical injuries, epigenetics, and gut microbiome leads to peritoneal membrane dysfunction that potentiate systemic peritoneal inflammation and fibrosis.
• Information highlighting the evolving paradigm and outlining the novel modifications in Peritoneal Dialysis solutions supporting dialysis modification by the putative treatment.
• Advances in knowledge about the potential pharmacological/stem cell therapy interventions on canonical/non-canonical pathways involved in peritoneal membrane fibrosis.
• Identifying novel targets and developing corresponding therapeutics offer an essential means of advancing new treatments for Peritoneal Fibrosis.

## 1 Peritoneal Fibrosis- an overview

Peritoneal dialysis (PD) is a well-known alternative to haemodialysis (HD) and a cost-effective method of renal replacement therapy in patients with end stage renal disease (ESRD), which is a global health burden (Thurlow et al., 2021). Approximately 11% of ESRD patients receive PD worldwide, making PD an important intervention in the management of ESRD (Thurlow et al., 2021; Balzer, 2020; Bello et al., 2022). Long-term PD induces peritoneal fibrosis in approximately 40% of the patients (Baroni et al., 2012). During PD, solute and water transport occur across the peritoneal membrane, which includes peritoneal mesothelial cells (PMCs), an interstitial matrix with fibroblasts and collagen, lymphatics, and a dense microvascular network (Morelle et al., 2023; Aroeira et al., 2007). Progressive damage to the peritoneal membrane during long-term PD impairs solute clearance, fluid balance, and membrane transport properties, often leading to ultrafiltration (UF) failure and technique dropout. Approximately 25% of PD patients experience severe fluid overload, primarily due to membrane failure, mechanical complications, and increased susceptibility to infections such as PD-related peritonitis (Htay et al., 2018; Morelle et al., 2021; Van Biesen et al., 2011; Lan et al., 2016). Long term exposure results in structural and functional alterations, including loss of peritoneal mesothelial cells (PMCs), submesothelial fibrosis, vasculopathy with luminal narrowing, angiogenesis, and persistent inflammation, all contributing to membrane dysfunction and PD failure (Williams et al., 2003). Therefore, the longevity of PD is limited and most PD patients eventually switch to Haemodialysis (HD) within a few years.

**FIGURE 1:**
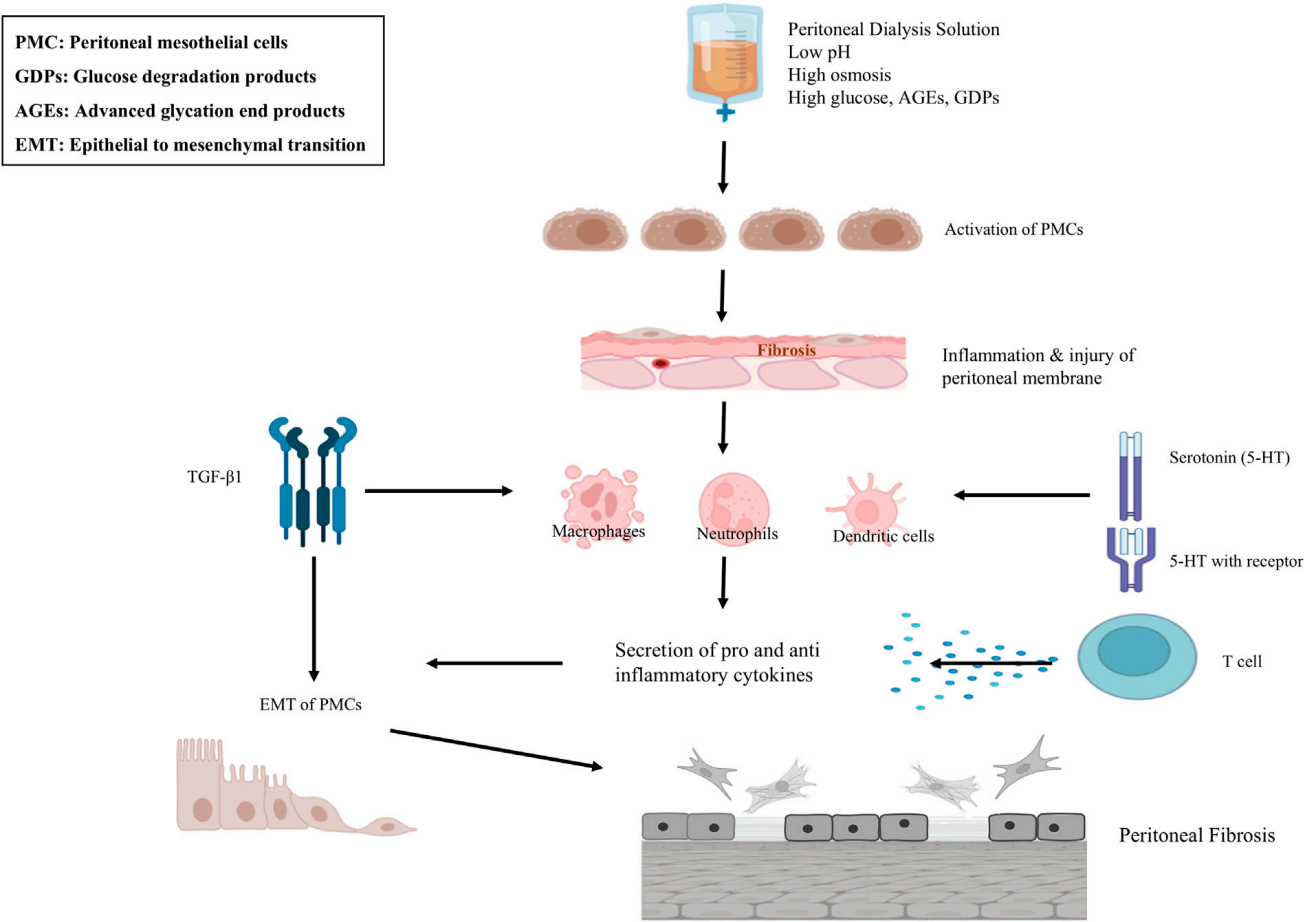
Mechanisms involved in peritoneal fibrosis. During long term PD peritoneal membrane is exposed to various insults that cause inflammation and injury such as, PMCs, ECs, macrophages and neutrophils produce various proinflammatory cytokines and growth factors. After activation, recognition process of bacterial pathogens occurs *via* TLRs, then after they undergo activation of the nuclear factor-kappa B (NF-kB) signalling pathway. This leads to the secretion of various inflammatory cytokines, including IL-6, IL-1β, IL-8, TNF-α, MCP-1, and MIP 2. These factors induce EMT process of PMCs, which results in fibroblast-like cells transformation that secrete ECM. They also elicit chronic inflammation and angiogenesis in the peritoneal cavity. The above processes contribute to the proliferation of fibroblasts and collagen deposition, which cause the thickening and stiffening of the peritoneum leading to peritoneal fibrosis. Abbreviations: PD, Peritoneal Dialysis; PMCs, Peritoneal Mesothelial Cells; ECs, Endothelial Cells; TLRs, Toll Like Receptors; IL, Interleukins; EMT, Epithelial to Mesenchymal Transition; ECM, Extracellular Matrix.

Acidic and hypertonic PD solutions trigger biochemical changes in the extracellular matrix and alter PMC phenotypes, promoting peritoneal fibrosis—the final stage of membrane remodeling (Terri et al., 2021; Zhou et al., 2016). A key mechanism in this process is epithelial-to-mesenchymal transition (EMT), where PMCs lose their epithelial characteristics and acquire a mesenchymal, fibroblast-like phenotype with increased motility and invasiveness (Wilson et al., 2020). The transformation of PMCs, fibroblasts into myofibroblasts is mediated by Transforming Growth Factor β1 (TGF-β1) (Wilson et al., 2020). TGF-β1 induces EMT transition primarily through the SMAD2/3 pathway, as well as non-canonical pathways including RAS/RAF/MEK/ERK, PI3K/AKT/mTOR, and STAT3 signalling (Wilson et al., 2020; Wilson, 2018). The above signalling pathways triggers inflammatory cascade leading to the production of numerous inflammatory cytokines, pro-fibrotic molecules, Interleukin (IL)-1ß, IL-6, tumour necrosis factor α (TNF-α) Monocyte Chemoattractant Protein-1 (MCP-1), Connective Tissue Growth Factor (CTGF), Platelet-Derived Growth Factor (PDGF) and Vascular Endothelial Growth Factor (VEGF) in the membrane (Balzer, 2020; Terri et al., 2021; Strippoli et al., 2016; Mutsaers et al., 2016), The above injurious agents lead to a life-threatening complication of long-term PD, encapsulating peritoneal sclerosis (EPS), occurring in 14%–15% of the patients (Terri et al., 2021; Strippoli et al., 2016). This inflammatory response cause degeneration of peritoneal membrane structure and function (Zhou et al., 2016; Liu et al., 2022; Fung et al., 2020; Kim et al., 2019; Budi et al., 2017). Figure 1 explains the induction of progressive peritoneal fibrosis during long-term exposure to bio-incompatible PD solutions.

The current comprehensive review provides information that assists in elucidating the recent advances in the pathophysiology involved in peritoneal fibrosis and explores the molecular mechanisms and pathways in treating or preventing the same. Therefore, in view of current medical requirements, herein we have discussed effective therapeutic approaches and pharmacological interventions selectively targeting the molecular activation of fibroblasts during fibrosis which warrant further investigations.

## 2 Methods

We searched PUBMED for that reported peritoneal membrane inflammation and fibrosis linked to long-term peritoneal dialysis, containing *in vitro* and *in vivo*, as well as preclinical and clinical studies. We limited the search to articles published in English and also included previous relevant research (1998–2025).

## 3 Mechanisms involved Peritoneal Fibrosis

Multiple lines of evidence point to the importance of mechanisms involved in the peritoneal membrane alterations described as EMT of PMCs and generation of myofibroblasts, which play a characteristic role in the subsequent functional deterioration of the peritoneal membrane (Terri et al., 2021; Strippoli et al., 2016). Peritoneal membrane injury can result in EPS, a serious complication of peritoneal fibrosis with potential fatal manifestation characterized by ultrafiltration failure, inflammation, severe peritoneal thickening, fibrin deposition, and calcification (Park et al., 2008). Plasma exudation from peritoneal microvessels causes fibrin deposition, which is the pathological feature of EPS (Kawaguchi et al., 2000; Honda and Oda, 2005). PMCs loss, impaired fibrinolysis, submesothelial myofibroblasts proliferation, collagen and AGE accumulation in the submesothelial layer are the main features of EMT in EPS (Wilson, 2018; Del Peso et al., 2008). At present, the pathogenesis of peritoneal fibrosis has not been fully elucidated. Factors such as activation of myo-fibroblasts, EMT of PMCS, biomechanical injuries, epigenetics and gut microbiota, bioincompatible PD fluid-induced sterile inflammation, peritonitis, peritoneal angiogenesis are all involved in the occurrence of peritoneal fibrosis.

### 3.1 Peritoneal Fibrosis: the fibroblasts switch and epithelial to mesenchymal transition of peritoneal mesothelial cells

**FIGURE 2:**
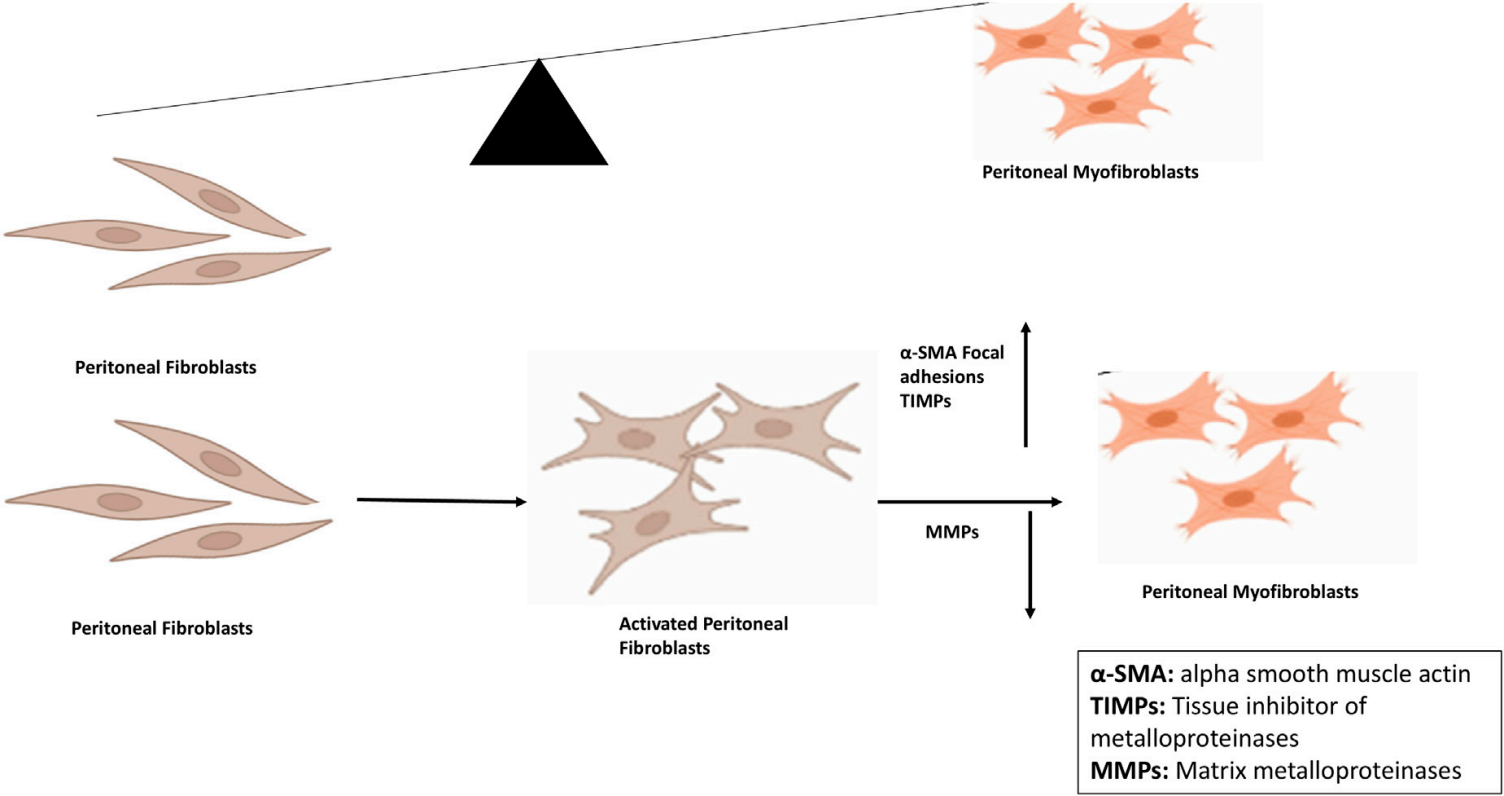
Peritoneal fibroblast-myofibroblast trans differentiation and its implications for peritoneal fibrosis. Conversion is the key process involved in peritoneal fibrosis in which expression of alpha smooth muscle actin as well as of focal adhesions is increased. After conversion, synthesis and secretion of extracellular matrix proteins take place leading to decreased expression of MMPs and increased expression of TIMPs. Abbreviations: MMPs, Matrix metalloproteinases; TIMPs, Tissue inhibitors of metalloproteinases.

Fibroblast activation is a normal component of wound healing; however, in PD patients, it becomes persistent and leads to the accumulation of activated myofibroblasts that alters the healthy structure and function of the peritoneal membrane, thus hindering the effective treatment (Strippoli et al., 2020; Pap et al., 2020). EMT, a complex biological process in the peritoneal membrane, involves the transformation of PMCs which lose epithelial phenotype (apical, basal polarity) and detach from characteristic basement membrane/ECM attachment, and acquire myofibroblast-like cells characteristics such as invasive ability and mesenchymal phenotype (Figure 2) (Wilson et al., 2020; Yáñez-Mó et al., 2003).

**FIGURE 3:**
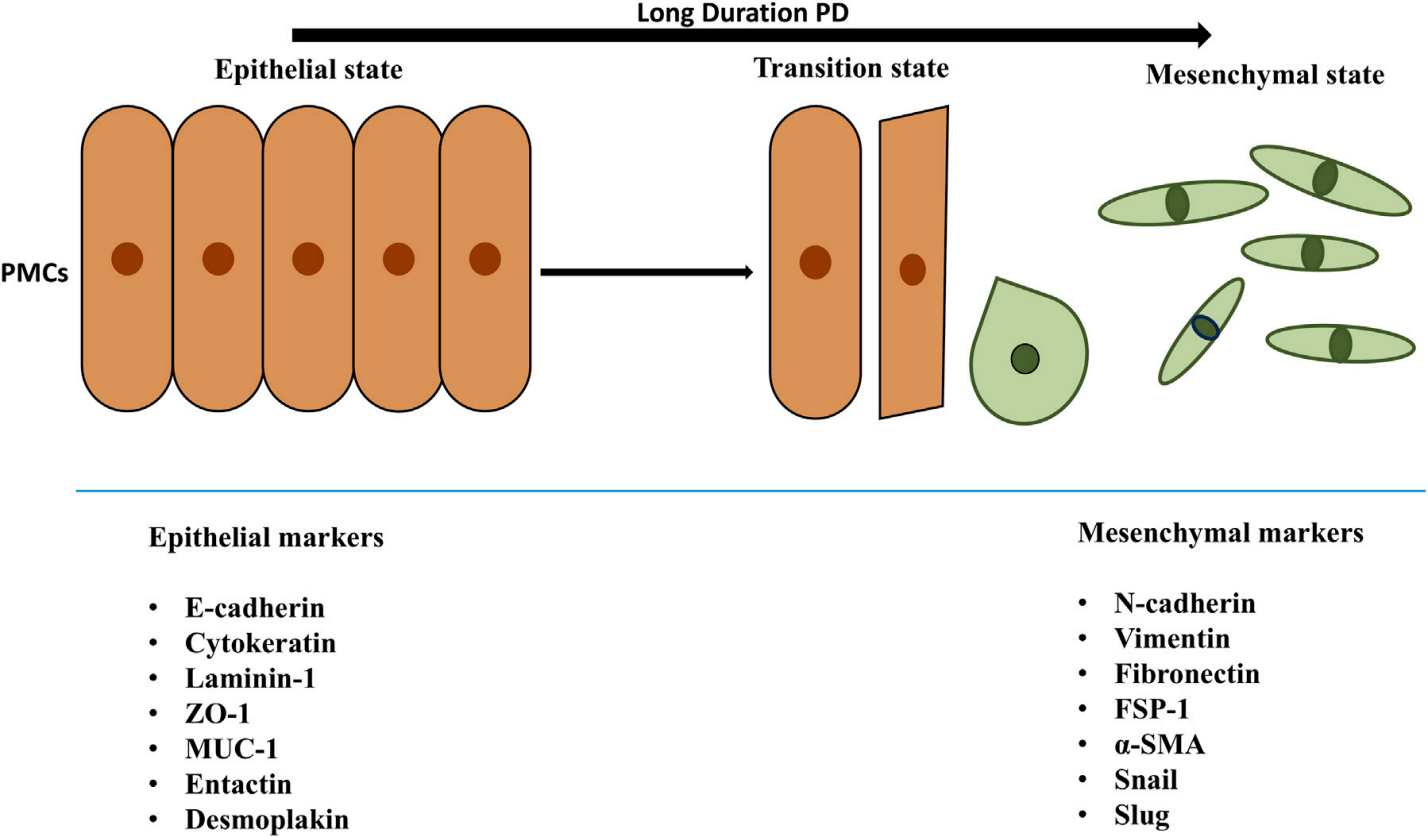
Transition of PMCs from epithelial to mesenchymal state. The epithelial and mesenchymal cell markers are mentioned in the above figure. Peritoneal mesothelial cells of PM explicit epithelial markers such as cytokeratin, ICAM1, E-cadherin. Also, during EMT leading mesenchymal cells are noted because of epithelial markers downregulation and upregulation of mesenchymal markers like α-SMA, fibronectin, vimentin, FSP-1. The epithelial and mesenchymal cell markers are mentioned in the above figure. Peritoneal mesothelial cells of PM explicit epithelial markers such as cytokeratin, ICAM1, E-cadherin. During EMT, mesothelial cells experience a decrease in the expression of epithelial markers, including E-cadherin, enhancing the expression of mesenchymal markers like α-SMA, fibronectin, vimentin, FSP-1. As a consequence, cells acquire invasive capacities and reach the sub-mesothelial stroma, where they produce extracellular matrix—but also inflammatory and angiogenic—factors, promoting peritoneal oxidative stress, inflammation and, finally, fibrosis, affecting peritoneal transport of water and solutes and resulting in ultrafiltration failure. Abbreviations: PD, Peritoneal Dialysis; PMCs, Peritoneal Mesothelial Cells; ZO-1, Zona Occludens 1; ICAM1, Intercellular Adhesion Molecule 1; MUC1, transmembrane glycoprotein mucin 1; α-SMA, Alpha Smooth Muscle Actin; FSP-1, Fibroblast Specific Protein -1.

Cellular trans-differentiation is a complex phenomenon that converts epithelial cells into mesenchymal cells. This biological process, called EMT, depends on the extracellular environment rather than the genome (Stone et al., 2016). EMT results in the loss of cell polarity and junctions and the gain of fibroblastic shape and invasiveness (Figure 3) (Stone et al., 2016). EMT occurs in both physiological (e.g., organogenesis, development, wound healing, and regeneration) and pathological (e.g., fibrosis, metastasis) conditions (Leggett et al., 2021). PMCs migrate and proliferate on the serosal lining (Tsai et al., 2018) and differentiate into myo-fibroblasts (EMT), which may promote fibrotic conversion (Sandoval et al., 2016) Changes in the gene expression and phenotype of PMCs, making them deposit more collagens and fibronectin and increasing their motility, which facilitate the development of peritoneal fibrosis (Strippoli et al., 2016).

EMT may play a pivotal role in the early decline of UF capacity, which constitutes the most critical functional impairment during the initial years of PD. A significant correlation has been established between UF efficiency and the dialysate-to-plasma (D/P) creatinine ratio, indicating a shared pathophysiological mechanism (Krediet, 2024; Davies, 2004). However, in many cases, the degree of UF impairment exceeds what would be anticipated based solely on D/P creatinine values (Krediet, 2024). This paradox—characterized by increased solute transport (elevated D/P creatinine) alongside diminished UF—is a hallmark of UF failure secondary to peritoneal fibrosis (Del Peso et al., 2008). The net consequence is fluid overload in PD patients, despite apparent solute equilibration, underscoring the dissociation between solute and water transport in the fibrotic peritoneal membrane.

In addition, during EMT, changes in cell membrane receptors, signaling molecules such as TGF-β1, Src, Hypoxia-inducible factor (HIF-1α), and cell morphology and behavior occurs (Wilson et al., 2020). EMT involves complex pathological cross-talk among PMCs, endothelial cells, immune cells, and resident fibroblasts (Strippoli et al., 2020). TGF-β1, is constitutively expressed by the PMCs and plays a major role in the maintenance of a transformed, inflammatory micro-environment in peritoneal membrane (Wilson, 2018). It enhances HIF-1α expression, which drives cell growth, extracellular matrix production and cell migration (Wilson, 2018). Importance of pro-fibrotic, TGF-β1 signalling during EMT of PMCs has been demonstrated by using TGF-β receptor inhibitor GW788388 on the EMT signalling pathway (Lho et al., 2021). Therapeutic Interventions targeting EMT process are detailed in Table 1.

**TABLE 1:** Pharmacological interventions in fibroblasts conversion/EMT/MMT.

Approaches	Mechanism of action(s)	Target(s)	PD model(s)	References
GW788388 (TGF-β1 Receptor Inhibitor)	TGF-βI, ALK4, ALK7, TGF-βII receptor inhibition, Inhibition of conversion of mesothelial cells phenotype to intermediatory cells phenotype during MMT, Improved peritoneal membrane integrity and function, reduced fibrosis and inflammation	TGF-βI, ALK4, ALK7, and TGF-βII receptors	HPMCs from omentum of biopsy proven patients with stomach cancer, Mice model of PF (0.1% CG and 15% ethanol dissolved in PBS were injected i.p. into mice every other day and oral gavage using 30% PEG400, 0.5% Tween 80, and propylene glycol in water (0.2 mL/body) was performed daily)	Wilson et al. (2020), Lho et al. (2021)
BMP-7	Blocks TGF-β1 driven MMT; Inhibition of conversion of mesothelial cells phenotype to intermediatory cells phenotype during EMT, reduced fibrosis and inflammation	TGF-βI/Smad signalling, Receptors of Glucose and Degradation Products	Rats were daily instilled with PD fluid for 5 weeks, HPMCs from omentum undergoing elective abdominal surgery, Combo model of PF and CKD with severe uremia in Wistar rats. PF induced by i.p. injections of CG, CKD by an adenine-rich diet	Loureiro et al. (2010), Yu et al. (2009), Silva et al. (2019)
Tamoxifen	Inhibits MMT, production of matrix components, preservation of fibrinolytic capacity of PMCs, decreased invasion capacity of PMS, suppression of EPS associated with PD, reduced peritoneal membrane thickness, improved peritoneal function for decreased solute transfer rate	Receptors of Glucose and GDPs, TGF-βI receptor, Snail signalling, MMT markers (fibronectin, type I-collagen, α-SMA, MMP-2), VEGF, Leptin, ESR1, H19 promoter and p300, VEGF-A	Combo model of PF and CKD with severe uremia in Wistar rats. PF induced by i.p. injections of CG, CKD by an adenine-rich diet, HPMCs obtained from omentum undergoing elective abdominal surgery. C57BL/6 mice daily instilled with 1.5 mL of standard PD fluid (4.25% glucose and buffered with lactate), HPMCs were cultured from PD effluent with dwell times ranging from 7 to 10 h	Wilson et al. (2020), Silva et al. (2019), Loureiro et al. (2010), Loureiro et al. (2013), Zhao et al. (2023)
Rapamycin (mTOR inhibitor)	Inhibits Endo-MT, partial MMT, reduced peritoneal membrane thickness, angiogenesis, improved peritoneal UF rate	VEGF-C, VEGF-D, TNF-α, TGF-βI, CD31and FSP1 in the submesothelial cell layer in the compact zone, Tubulin	C57BL/6 mice daily instilled with 2 mL of standard PD fluid composed of 4.25% glucose and buffered with lactate, HPMCs obtained from omentum undergoing elective abdominal surgery	González-Mateo et al. (2015)
β-catenin inhibitor (ICG-001) WNT antagonist (DKK-1)	Blocks MMT, Reduced Angiogenesis, mitigation of PMCs injury, better membrane function	WNT1, WNT2, WNT4, VEGF, E-Cadherin, β-catenin, GSK-3β, c-MYC, MMP7, SFRP, DKK	WNT1 protein expression in peritoneal effluents of PD patients and WNT gene expression in PMCs from PD patients, C57BL/6 mice i.p. injection of AdTGF- β along with elements of the WNT signalling pathway, HPMCs cell line HMrSV5	Padwal et al. (2018)
Astragalus	Inhibits EMT, inflammation, fibrosis	Wnt/β-catenin signalling, MMT Markers (E Cadherin, α-SMA, Vimentin), GSK-3β, Smad7	HPMCs cell line HMrSV5, rat model of PD i.p. injection of standard PD fluid composed of 4.25% glucose and buffered with lactate at 100 mg/kg/day for 35 days	Yu et al. (2018)
Curcumin	Suppresses EMT, inflammation, fibrosis, reduced migration and invasion of PMCs	MMT Markers (E Cadherin, α-SMA), ECM proteins (Type 1 collagen and Fibronectin), TGF-βI, phosphorylated-TAK1, JNK and p38 pathway	HPMCs cell line HMrSV5	Zhao et al. (2019)
Metformin	Block mtDNA release and inhibits EMT, reduced angiogenesis, inflammation, and fibrosis, oxidative stress	Smad2/3 and MAPK, GSK-3β phosphorylation, β-catenin and Snail signalling	Primary HPMCs and animal model of PD. Dialysis solution of 25 mL (4.25% Dianeal^®^, pH 5.2) was infused twice daily, 7 days per week for 8 weeks	Shin et al. (2017)
Asiaticoside	Inhibits MMT, inflammation, fibrosis and oxidative stress, reduced migration and invasion of PMCs, better membrane function	TGF-βI/Smad signalling, Nrf2/HO-1 signalling	HPMCs cell line (HMrSV5)	Zhao et al. (2020)
Saikosaponin D	Inhibits MMT, inflammation, fibrosis	TGFβ1/BMP7/Gremlin1/Smad 1/5/8 signalling	Male SD rat 5/6 nephrectomy model and received 15 mL of 4.25% dialysate was injected ip every day, HPMCs cell line HTX2481	Ruiqi et al. (2021)
Exosomal lnc-CDHR derived from human umbilical cord MSCs	Reduced EMT, inflammation, fibrosis	MMT Markers (α-SMA, Vimentin, E-cadherin), PTEN, and AKT/FOXO3a	HPMCs cell line (HMrSV5)	Jiao et al. (2023)
Sirtuin1 (SIRT1)	Reduced EMT, inflammation, fibrosis, protein matrix deposition, peritoneal membrane failure during SIRT1 upregulation	TGF-β1/Smad3 signalling, Col1a1, FN, a-SMA, Snail, IL-6, IL-1β, MCP-1	C57BL/6 mice PD model of peritoneal fibrosis was induced by daily 3 mL i.p. injection of 4.25% PD fluid (Dianeal containing 4.25% glucose) for 4 weeks	Guo et al. (2021)
Tubastatin A (HDAC6 inhibitor)	Reduced EMT, inflammation, fibrosis, protein matrix deposition, inhibition of M2 macrophages polarization	TGF-β1/Smad, IL4/STAT6, STAT3 and PI3K/AKT signalling, MMP-2, MMP-9	Mouse model of PF i.p. injection of 0.1% CG (10 mL/kg) dissolved in saline every other day for 21 days	Shi et al. (2022a)
MS-275 (HDAC1-3 inhibitor)	MMT reversal promotion, inhibition of PMCs migration and invasion	TGFβRI mRNA-targeting miRNAs (miR-769-5p), SMAD2/3 and PAI-1 expression, WT1	Effluent-derived MCs isolated from PD patients, human mesothelial cell line MeT‐5 A	Rossi et al. (2018)
S3I-201 (STAT3 inhibitor)	Reduced EMT, angiogenesis, inflammation, fibrosis, macrophage infiltration	STAT3/HIF-1α signalling, MMT Markers (Collagen I Fibronectin, α-SMA, E-cadherin)	Human mesothelial cell line MeT‐5A, HPMCs obtained from the effluent of PD patients	Yang et al. (2021)
Vitamin D receptor activation	Reduced EMT, proliferation of myofibroblasts, macrophage infiltration, expression of TGF-β, MCP-1, VEGF	MMP Proteins (type 1 collagen, fibronectin), Th17 cells, COX-2/PGE2 axis, STAT3/RORγt expression	HPMCs, Rat model of PF infused with a conventional 4.25% PD solution	Marchant et al. (2020), Kang et al. (2014)

Abbreviations: ALK4/7, Activin Receptor like Kinase 4/7; HPMCs, Human Periotneal Mesothelial Cells; CG, chlorhexidine gluconate; MGO, methylglyoxal; PBS, phosphate buffered saline; i. p., intraperitoneal; ; PEG, polyethylene glycol; BMP-7, Bone Morphogenic Protein; FSP-1, Fibroblast Specific protein 1; (DKK)-1, Dickkopf-related protein; SFRP, Secreted frizzled-related proteins; GSK-3β, Glycogen Synthetase Kinase; AdTGF, β; Adenovirus expressing TGF-β; Nrf-2, Nuclear factor erythroid 2-related factor 2; PI3K, Phosphatidylinositol 3-kinase; NF-κB, Nuclear Factor-κB; PAI-1, Plasminogen Activator Inhibitor-1; RORγt, Retinoid related orphan receptor γt; TAK1, TGF-β; activated kinase 1; MSCs, Mesenchymal Stem Cells; WT1, Wilms’ tumor 1 transcription factor; GSK-3β, Glycogen Synthetase Kinase; Nrf-2, Nuclear factor erythroid 2-related factor 2; NF-κB, Nuclear Factor-κB; MCP-1, Monocyte Chemoattractant Protein-1; PGC1α, PPARγ; coactivator-1α; HIF-1α, Hypoxia-inducible factor-1α; ACE, angiotensin converting enzyme; MR, mineralocorticoid receptor; 11b-HSD2, 11b-hydroxysteroid dehydrogenase type 2; CYP11B2, Aldosterone synthase; BRG-1-Brahma related gene-1; ZO-1, Zonula Occludens-1; SAHA, suberoylanilide hydroxamic acid; 3-DZNeP, 3-deazaneplanocin A; EZH2, Histone methyltransferase enhancer of zeste homolog 2; AP-1-Activator Protein-1; PDTC, pyrrolidine dithiocarbamate; SGLT, Sodium-Dependent Glucose Transporters; MTAC, mass transfer area coefficients; 8-OHdG, 8-Hydroxydeoxyguanosine; MDA, malondialdehyde; BET, bromodomain and extra terminal domain; Cat, Catalase; Hmox1, Heme Oxygenase 1; Sod1-Superoxide Dismutase 1; MSCs, Mesenchymal Stem Cells; HSCs, Haematopoietic Stem Cells; BM, bone marrow; GFP, green fluorescent protein.

### 3.2 Biomechanical Lesions

Mechanotransduction, the process of transforming mechanical signals into biochemical events, along with extracellular biochemical factors, regulates various cellular functions and is crucial during development, physiological and pathological conditions (Terri et al., 2021; Strippoli et al., 2020; Orr et al., 2006; Santos and Lagares, 2018). Biomechanical alterations of the ECM, including increased ECM rigidity or forces of varying strengths and dynamic characteristics, modulate form and the functions of cells with an inevitable impact on the cellular behavior and therefore drive distinct fibrotic signaling pathways (Orr et al., 2006; Santos and Lagares, 2018; Chen, 2008). PD requires the infusion of large amounts of PD solutions into the peritoneal cavity, which exposes the peritoneal membrane to biomechanical forces. These forces include mechanical stretch of PMCs and augment intra-abdominal pressure. Additionally, reports state that abdominal surgeries can drive fibrotic conversion pertaining to the vast majority of adhesion myofibroblasts which arise from surface mesothelium followed by EMT process which may induce biomechanical injury to the peritoneal membrane leading to peritoneal adhesions formation (Sandoval et al., 2016; Zindel et al., 2021). Proliferation and migration of mesothelial cells is promoted by receptor tyrosine kinases of the ERBB family, Epidermal Growth Factor Receptor (EGFR), which is documented to be involved in post-surgical peritoneal adhesions (Zindel et al., 2021). Mechanical injury and hypoxia identified by injured surface mesothelium expressing podoplanin (PDPN)/mesothelin (MSLN) and upregulation of hypoxia-inducible factor 1 alpha (HIF1α) are involved in the fibrotic process and the development of peritoneal adhesions suggested in an *in vivo* study (Tsai et al., 2018).

### 3.3 Epigenetics and Gut Microbiome: the dynamic duo behind peritoneal fibrosis

Peritoneal fibrosis, which is caused by inflammation, infection, or long-lasting dialysis, leads many patients to discontinue PD, however, its mechanism is unclear. Epigenetic shifts are involved in peritoneal fibrosis, and emerging evidence indicates that epigenetic interventions could help ward off and treat peritoneal fibrosis in practice (Tsai et al., 2018; Wang et al., 2021). Epigenetic regulation in peritoneal fibrosis is complicated, mainly affecting the changes of signalling molecules, transcriptional factors, and genes (Tsai et al., 2018; Wang et al., 2021). The primary epigenetic transformations include, changes in the expression and activity of genes (such as DNA methylation, histone modifications), and different types of non-coding RNA molecules (such as microRNAs (miRNAs), long non-coding RNAs, and circular RNAs) can also play a role in the development of peritoneal fibrosis (Tsai et al., 2018; Sandoval et al., 2016; Wang et al., 2021; Guo et al., 2020). HDAC inhibitors are discussed in Table 1 and 2. The development of peritoneal fibrosis (PF) is significantly influenced by non-coding RNAs, particularly microRNAs (miRNAs) and long non-coding RNAs (lncRNAs). miRNAs are known to regulate key molecular pathways involved in PF and are increasingly being recognized as potential diagnostic biomarkers and therapeutic targets (Sandoval et al., 2016; Guo et al., 2020; Huang et al., 2023). For instance, miR-15a-5p is found to be downregulated in long-term PD patients, suggesting a role in fibrosis progression. Beyond this, a range of other miRNAs—including miR-199a-5p, miR-214-3p, miR-153-3p, miR-129-5p, miR-21, miR-30a, miR-145, miR-30b, miR-200, miR-302c, miR-34a, and miR-29b—have been reported to interact with hypoxia-inducible factor-1α (HIF-1α), a key regulator in fibrotic signaling (Sandoval et al., 2016; Guo et al., 2020). Concurrently, growing evidence suggests that lncRNAs also contribute to the pathogenesis of peritoneal fibrosis, although their precise mechanisms remain under investigation. As research in this field advances, it is crucial to identify practical approaches for targeting these non-coding RNAs to improve peritoneal fibrosis diagnosis and treatment.

**TABLE 2:** Pharmacological interventions in inflammation/membrane thickness/oxidative stress/angiogenesis/fibrosis.

Approaches	Mechanism of action(s)	Target(s)	PD model(s)	References
RhoA/Rho-kinase inhibitors (Fasudil and Y-27632) AP-1 inhibitor (Curcumin)	Normalized histopathological features, prevent ultrafiltration failure and preserved peritoneal membrane function, decrease in membrane thickness as well as motility and invasion capacity in AGEs treated HPMCs	RhoA/Rho-kinase signalling and activating protein-1 (AP- 1), TGF-βI receptor, MMT markers (fibronectin, type I-collagen, α-SMA, Vimentin, N-cadherin, E-cadherin), Receptors of Glucose and Degradation Products	HPMCs cell line (HMrSV5), rat PF model generated by daily i.p. injection of DIANEAL (4.25% dextrose-monohydrate PD fluid) at 100 mL/kg for 4 weeks	Wang et al. (2018)
Sildenafil and SB204741 (5HT_2B_ receptor) combination	Restoring of MMP2/TIMP1 ratio and increased IL-10 levels. Decreased collagen production, α-SMA, and other pro-inflammatory cytokines. Decreased inflammation and fibrosis	Pro and anti-fibrotic genes (*COL1A1*, *COL1A2*, *ACTA2*, *CTGF*, *FN1*, *TGFB1*, *MMP2/TIMP1*). Pro and Anti-inflammatory cytokines ((IFN-γ, IL-4, IL-17, IL-1β, IL-6, TNF-α, TGF-β1, IL-10)	HPFBs isolated from parietal peritoneum biopsy and incubated in DMEM/10% FBS/1% penicillin/streptomycin/amphotericin-B at 37° C	Chaturvedi et al. (2024)
Arctigenin	Reduced inflammation, decreased ROS levels and fibrosis	TGF-βI receptor, MMT markers (α-SMA, vimentin, fibronectin, E-cadherin), PAI-1, AMPK, IkBa, NF-kB	HPMCs cell line	Jin et al. (2019)
Niao Du Kang Mixture	Maintain the morphological structure of PMCs, decreased peritoneal membrane thickness and preserve its function, mitigation of PMCs injury, reduced inflammation and fibrosis	Wnt/β-catenin signalling, MMT markers (E-cadherin, α-SMA, Wnt-1, collagen I, β-catenin, and LEF-1)	Rat PF Model. One week after modelling, 4.25% glucose peritoneal dialysate (30 mL/kg) was injected i.p. once daily for 28 consecutive days	Huang et al. (2022)
C75 (CPT1A activator)	Reduced mtROS generation, increased mtDNA number, reduced inflammation, fibrosis and oxidative stress	FAO, CPT1A, SNAIL1, SNAIL2, ZEB2, FN1, COL1A1, UPK3B (marker of mesothelial cells), Smad3, PGC1α	HPMCs from PD patients’ effluent C57BL/6 PD model of peritoneal fibrosis was induced by daily i.p. injection of 4.25% PD fluid (DIANEAL containing 4.25% glucose) at 100 mL/kg body weight for 6 weeks	Su et al. (2023)
Salvianolic acid A	Improved the elicited peritoneal fibrosis response, reduced thickening of the sub-mesothelial compact zone, inflammation, fibrosis and oxidative stress	GSK3β/NFκB phosphorylation and Nrf2, MMT Markers (Vimentin and PAI-1), TNF-α, IL-1β, and MCP-1	C57BL/6 PD model of peritoneal fibrosis was induced by daily 3 mL i.p. injection of 4.25% PD fluid (Dianeal containing 4.25% glucose).	Zhou et al. (2022)
Tetramethylpyrazine	Reduced angiogenesis, migration, inflammation, fibrosis and peritoneal membrane injury	VEGF/Hippo/YAP signalling, p-ERK, p-P38, and p-Akt	HPMCs cell line (HMrSV5), HPVECs, C57BL/6 mice PD model of peritoneal fibrosis was induced by daily i.p. injection of 4.25% PD fluid (Dianeal containing 4.25% glucose) at 10 mL/kg/day for 30 days	Zhu et al. (2021)
Baicalein (5,6,7-trihydroxyflavone)	Reduced inflammation, fibrosis and peritoneal membrane thickness	AGE-RAGE signalling, MMP2, BAX, ADORA3, HIF1A, PIM1, CA12, and ALOX5, MMT markers (fibronectin, type I-collagen, a-SMA)	Mice PD model of peritoneal fibrosis was induced by daily i.p. injection of 4.25% PD fluid (Dianeal containing 4.25% glucose)	Lu et al. (2023)
Spironolactone (Aldosterone receptor antagonist)	Reduced inflammation, fibrosis, peritoneal macrophages secretion, protection of peritoneal membrane function	MR, 11b-HSD2, CYP11B2, MCP-1, FN, TGF-β1, JNK pathway, CD-4 and ED-1-positive cells	Rat PD model of peritoneal fibrosis was induced by daily i.p. injection of 4.25% PD fluid (Dianeal containing 4.25% glucose) in a 4-day interval for a period of 7 days	Zhang et al. (2014)
Captopril, Quinapril (ACE inhibitors)	Reduction in parietal and visceral peritoneum thickness, EPS and amelioration of fibrotic change in parietal peritoneum	Parietal and visceral peritoneum cells, subserosal fibrotic matrix, subserosal large collagen fibers, and subserosal fibroblast proliferation	Non-uremic Wistar rats received a peritoneal infusion of 10 mL/100g of PD solution glucose 4.25% on a daily basis, C57BL/6 mice model of peritoneal fibrosis was induced by daily i.p. injection of 0.3 mL of SH solution which consists of 0.1% chlorhexidine gluconate	Schu et al. (2013), Sawada et al. (2002)
Micheliolide	Reduced inflammation, fibrosis and oxidative stress	BRG-1- H3K14ac complex, TGF-β1/Smad signalling, MMT markers (Fibronectin and type 1 collagen)	C57BL/6 mice PD model of peritoneal fibrosis was induced by daily 3 mL i.p. injection of 4.25% PD fluid (Dianeal containing 4.25% glucose) for 4 weeks, HPMCs cell line HMrSV5	Li et al. (2023)
SAHA (Nonspecific HDAC inhibitor)	Reduced submesothelial thickness, type III collagen accumulation, increased histone acetylation, suppressed inflammation and angiogenesis	FSP1, α-SMA, Smad 2/3, *Col1a1*, *FN1*, *CTGF*, H3K9, BMP-7	Mouse model of PF induced by i.p. 0.1% CG	Yu et al. (2018), Io et al. (2015)
BIX01294 (H3K9 histone methyltransferase G9a inhibitor)	Decreased submesothelial zone thickness, fibrosis, infiltration of monocytes, peritoneal membrane function, ECM proteins	α-SMA, CD68, TGF-β1, and H3K9me1, ZO-1, G9a, type I and III collagen	Mouse model of PF induced by MGO, HPMCs	Zhao et al. (2019), Maeda et al. (2017)
Sinefungin (H3K4 methyltransferase SET7/9 inhibitor)	Decreased submesothelial zone thickness and PMCs accumulation, fibrosis, infiltration of monocytes, peritoneal membrane function, ECM proteins	SET7/9, α-SMA, fibronectin, ZO-1, α-tubulin, H3K4me1, H3, *ACTA2*, *Col1A2*, *CTGF*, *PAI-1*	C57/BL6 mouse model of PF induced by MGO, HPMCs	Shin et al. (2017), Tamura et al. (2018)
3-DZNeP (EZH2 inhibitor)	Attenuates fibrosis, EMT, improves membrane dysfunction. Reduces inflammation, lymphocyte and macrophage infiltration, angiogenesis, apoptosis, PMCs migration	EZH2, H3K27me3, p-EGFR, p-Src/Src, p-ERK1/2/ERK1/2, p-STAT3/STAT3, p- Smad3/Smad3. Notch1, MMP2/9, Histone H3, Cleaved caspase 3, α-SMA, type I collagen, fibronectin, E-Cadherin, p-NF-κB, TGF-βRI, Smad7, CD68, CD31, VEGF, EGFR	HPMCs, EZH2-KO C-57/BL6 mice model of PF induced by CG or PD fluid	Shi et al. (2022b)
Soluble TLR2 (sTLR2) (TLR inhibitor)	Reduced inflammation, fibrosis development, suppressing pro-fibrotic gene expression, pro-inflammatory cytokine production, reduced leukocyte/neutrophil recruitment, recovery of Treg cell levels and increased Treg:Th17 ratio	TLR2/4, Hsp70, hyaluronan, IL-α, IL-β, CXCL8/IL-8, CXCL10/IL-10, ERK1/2 phosphorylation, IκB-α	Peritoneal leukocytes and mesothelial cells, C57BL/6 mice were instilled twice daily for 40 days with 2 mL standard 4.25% glucose solution	Raby et al. (2018)
Peroxisome Proliferator-Activated Receptor-γ (PPAR-γ) agonist PTDC (NF-κB inhibitor) SP600125 (AP-1 inhibitor)	Reduced angiogenesis, inflammation, fibrosis, TGF-β, ECM protein deposition; regulates T-cell-mediated peritoneal membrane protection; inhibits NF-κB, AP-1	Receptors of Glucose and Degradation Products, MMP Proteins (type 1 collagen, fibronectin), NF-κB and AP-1 signalling pathway, Th17/Treg cells, STAT3/RORγt expression	PMCs from surgically resected omentum of rat	Zhou et al. (2013)
Chemokine (C-C motif) ligand 8 receptor inhibitor (R243)	Reduced EMT, peritoneal inflammation, fibrosis, apoptosis	CCL8 and FN mRNA expression, TNFr1, IL-1b, IL-6, ICAM-1, p65, pp65, α-SMA, periostin, p53, β-galactosidase, and CCR2 protein expression	HPMCs, Mice model of PF injected with CG (0.2 mL i.p.) for 4 weeks	Lee et al. (2023)
Cationized gelatin microspheres (CGMs) containing Hepatocyte Growth Factor (HGF) expressing plasmids	Suppression of thickening of submesothelial compact zone, reduction in myofibroblasts proliferation, mitigate peritoneal hyperpermeability	HGF levels, type III collagen, TGF-β, α-SMA expression, dilute/serum ratio of creatinine (D/S Cr) ratio was observed	C57BL6/6J mice model of PF injected with CG (0.05%) dissolved in 0.2 mL of saline 3 times a week for 3 weeks	Obata et al. (2023)
LY294002 (PI3K) inhibitor Rapamycin (mTOR) inhibitor	Alleviate PF, Inhibition of ROS, 8-OHdG levels, MDA, Reduced membrane thickening, alleviated EMT	ROS, 8-OHdG levels, MDA, Glutathione Peroxidase Glutathione, ZO-1, FSP1, α-SMA, autophagy-related proteins LC3-II/I, p62, and beclin-1, PI3K/AKT/mTOR signalling pathway	PMCs culture from high-glucose (HG)-induced PF rat model	Jia et al. (2022)
SGLT_2_ inhibitor (Dapagliflozin)	Abrogation of PD fluid-induced SGLT2 transcriptional upregulation by i.p. dapagliflozin, Reduced Peritoneal Fibrosis and Ultrafiltration Failure, Reduced Submesothelial Microvessel Density, Abrogates Proinflammatory Signaling	SGLT1 and SGLT2, PET, D/D_0_ glucose ratio, dialysate-to-plasma ratios (D/P), MTAC for creatinine and urea, CD31, MCP-1, TNF-α, IL-6	Human peritoneal biopsies, C57BL6/6J mice model of PF exposed with 2.0 mL of standard PD fluid composed of 4.25% glucose and buffered with lactate, HPMCs, immortalized MPMCs, murine peritoneal macrophage cell line RAW264.7	Balzer et al. (2020)
EGFR inhibitor (Gefitinib)	Attenuation of Development of Peritoneal Fibrosis, Deposition of ECM, Activation of Fibroblasts, Suppression of Production of Inflammatory Cytokines and Infiltration of Macrophages, Attenuation of Angiogenesis	EGFR, Smad3, STAT3, NF-kB, TGF-β, TNF- α IL- β1, IL-6, MCP-1, collagen, α-SMA expression	Rat model of PF injected with 0.1% CG i.p., HPMCs	Wang et al. (2016)
BET Inhibitor (JQ1)	Reduced membrane thickness, inflammatory cells infiltration, peritoneal fibrosis, inflammation, oxidative stress, decreased gene overexpression of proinflammatory/profibrotic markers	NF-κB, IκBα, Fibronectin, Smad2/3, *IL-1*β, *TNF-*α, *Cat*, *Hmox1*, *Sod1*, *CCL2*, *CCL5*, *CXCL10*, *IL-6*, NRF2, NADPH Oxidase (NOX1/4)	C57BL/6 mice model of PF induced by 0.1% CG i.p., human mesothelial cell line MeT‐5A, Human-effluent-derived primary MCs	Marchant et al. (2023)
BM-MSCs and HSCs/AD-MSCs	Settlement and Differentiation of BM-MSCs and HSCs regenerate to MCs for remodelling of peritoneal membrane/AD-MSCs attenuate PF and maintain integrity by immunoregulation, modulating macrophage polarization *via* IL-6	Sca-1 or -c-Kit and GFP antibodies/M1 and M2 Macrophages, IL-6, Arg-1 expression	BM cells from GFP transgenic mice transplanted into naïve C57 B l/6 mice (PF model injected with CG)/Commercial human ADSCs, Rat Model of PF induced by Methylglyoxal	Sekiguchi et al. (2012)

Abbreviations: ALK4/7, Activin Receptor like Kinase 4/7; HPMCs, Human Periotneal Mesothelial Cells; CG, chlorhexidine gluconate; MGO, methylglyoxal; PBS, phosphate buffered saline; i. p., intraperitoneal; PEG, polyethylene glycol; BMP-7, Bone Morphogenic Protein; FSP-1, Fibroblast Specific protein 1; (DKK)-1, Dickkopf-related protein; SFRP, Secreted frizzled-related proteins; GSK-3β, Glycogen Synthetase Kinase; AdTGF, β; Adenovirus expressing TGF-β; Nrf-2, Nuclear factor erythroid 2-related factor 2; PI3K, Phosphatidylinositol 3-kinase; NF-κB, Nuclear Factor-κB; PAI-1, Plasminogen Activator Inhibitor-1; RORγt, Retinoid related orphan receptor γt; TAK1, TGF-β; activated kinase 1; MSCs, Mesenchymal Stem Cells; WT1, Wilms’ tumor 1 transcription factor; GSK-3β, Glycogen Synthetase Kinase; Nrf-2, Nuclear factor erythroid 2-related factor 2; NF-κB, Nuclear Factor-κB; MCP-1, Monocyte Chemoattractant Protein-1; PGC1α, PPARγ; coactivator-1α; HIF-1α, Hypoxia-inducible factor-1α; ACE, angiotensin converting enzyme; MR, mineralocorticoid receptor; 11b-HSD2, 11b-hydroxysteroid dehydrogenase type 2; CYP11B2, Aldosterone synthase; BRG-1-Brahma related gene-1; ZO-1, Zonula Occludens-1; SAHA, suberoylanilide hydroxamic acid; 3-DZNeP, 3-deazaneplanocin A; EZH2, Histone methyltransferase enhancer of zeste homolog 2; AP-1-Activator Protein-1; PDTC, pyrrolidine dithiocarbamate; SGLT, Sodium-Dependent Glucose Transporters; MTAC, mass transfer area coefficients; 8-OHdG, 8-Hydroxydeoxyguanosine; MDA, malondialdehyde; BET, bromodomain and extra terminal domain; Cat, Catalase; Hmox1, Heme Oxygenase 1; Sod1-Superoxide Dismutase 1; MSCs, Mesenchymal Stem Cells; HSCs, Haematopoietic Stem Cells; BM, bone marrow; GFP, green fluorescent protein.

Recent study gives a rational basis and underscores the likely role of gut microbiota dysbiosis in peritoneal fibrosis (Lho et al., 2021; Stepanova, 2023). Compared to healthy controls, PD patients had less *Actinobacteria* and *Firmicutes* in their faecal microbiota, with a notable decrease in *Bifidobacterium* and *Lactobacillus*, and more *Pseudomonas aeruginosa*, according to a study by Wang et al. (2012). By lowering the pH through the production of acetic acid and lactic acid, *Bifidobacterium* and *Lactobacillus* influence the host positively, either by preventing the growth of pathogenic bacteria or by competing with them for nutrients and adhesion sites (Wang et al., 2012). ESRD induced alterations in the gut microbiota compromise the intestinal barrier and facilitate the translocation of microbial components and endotoxins into the systemic circulation and peritoneal cavity. *Dorea*, *Clostridium*, and *SMB53* are related to chronic low-grade inflammation, oxidative stress, and immune responses within and beyond the peritoneum, peritonitis in ESRD patients on PD (Wang et al., 2012; Stadlbauer et al., 2017; Luo et al., 2021).

A study revealed, in PD patients, uremic toxins such as indoxyl sulfate (IS) and p-cresol sulfate (PCS) accumulate due to increased gut permeability and incomplete clearance and increase the host’s susceptibility to pathogen invasion. This, along with catheter use and dietary restrictions, disrupts the intestinal microenvironment, promoting pathogenic bacteria and reducing beneficial short chain fatty acids (SCFAs)-producing microbes. Consequently, PD patients exhibit a higher relative abundance of *Proteobacteria* in their gut microbiota (Simões-Silva et al., 2018). *Proteobacteria*, a potential marker of gut dysbiosis, shows similar abundance in PD patients and their healthy family members. This suggests that long-term hospitalization, rather than dialysis alone, may contribute to gut microbiome alterations in PD patients (Teixeira et al., 2023).

In PD patients, gut microbiota differs in both taxonomic composition and metabolic function. Those with longer dialysis duration, higher peritoneal glucose exposure, and reduced residual renal function show distinct microbiome profiles and significantly lower fecal levels of SCFAs, including isobutyric and isovaleric acids (Jiang et al., 2021). Identifying these alterations can provide valuable insights for clinicians, enabling early and targeted interventions to reduce complications and mortality risk in patients with ESRD. A study documented, patients on PD had more pathogenic and less beneficial species in their faecal microbiome, which altered the predicted metagenome functions, compared to controls (Stadlbauer et al., 2017).

In recent years, Probiotics, prebiotics, and synbiotics have been reported to reduce uremic toxins such as endotoxins and p-cresol in PD patients. These interventions may also lower mortality rates associated with long-term PD. Additionally, they have been shown to improve gastrointestinal symptoms and enhance the quality of life in PD patients (Li et al., 2025).

Probiotic capsules containing *Bifidobacterium longum*, *Lactobacillus bulgaricus*, and *Streptococcus thermophilus* have been shown to enhance gastrointestinal absorption and digestion (Li et al., 2025). They also help reduce inflammatory markers, such as C-reactive protein (CRP) and Interleukin-6 (IL-6) (Pan et al., 2021). Elevated IL-6 levels in PD patients are associated with malnutrition and altered peritoneal small solute transport rates, which can increase mortality. A randomized controlled trial (RCT) noted that PD patients improved serum indoxyl sulfate (IS) levels by consuming 21 g/day of unripe banana flour. Insulin-type fructan decreases *Bacteroides thetaiotaomicron*, the IS-producing bacterium, by inhibiting its tryptophanase activity. This leads to reduced IS production in the gut of these patients (de Andrade et al., 2021). In severe cases of uremic toxicity related to p-cresol or para-cresol sulfates, synbiotics can promote the growth of *Bifidobacterium bifidum* strains and increase *Lactobacillus* abundance in the gut. This intervention effectively reduces intestinal p-cresol levels (Stuivenberg et al., 2022). Although clinical research on gut microbiomes in PD patients remains limited in scale, further studies may validate the use of probiotics, prebiotics, and synbiotics. These interventions have the potential to improve the gut microbiome and reduce complication rates in PD.

## 4 Pathophysiology behind Peritoneal Membrane deterioration

PD uses the peritoneal membrane as an organ to filter the blood. Chronic exposure of PD solutions induces low-grade inflammation in the peritoneal cavity, which can impair the peritoneal membrane and compromise its function as a dialyzer. However, inflammation can damage the peritoneal membrane over time, causing it to lose its function. Inflammation also triggers peritoneal remodelling and angiogenesis, which are major events that alter the structure and blood vessels of the peritoneal membrane (Catar et al., 2022).

### 4.1 PD solutions and Chronic Peritoneal Inflammation

Chronic Peritoneal inflammation is considered as an important event during the pathogenesis of PF (Zhang et al., 2017). Peritoneal injury leads to the activation of signal transducer and activator of transcription 3 (STAT3) and nuclear factor kappa-B (NF-κB), which also promotes the release of multiple proinflammatory cytokines/chemokines (Shi et al., 2021). Peritoneal mesothelium-derived CXC chemokine ligand 1 (CXCL1), a chemokine, associates with peritoneal micro vessel density in uremic patients undergoing PD. Thus, CXCL1 and its receptors may be novel targets for therapeutic intervention to prolong PD therapy (Catar et al., 2022). Other pharmacological interventions targeting receptors involved in inflammation in the peritoneal membrane dysfunction culminating to fibrosis are detailed in Table 2.

A major factor limiting the chronic use of PD remains peritoneal membrane failure due to prolonged exposure to bioincompatible PD solutions leading to the fibrosis of the membrane (Cho et al., 2014). Recent evidences have tried to explain the mechanisms linking inflammation–either infection-induced (peritonitis) or sterile (bioincompatible PD solutions) – involved with the cellular stress and membrane injury in the pathogenesis and regulation of PD related peritoneal fibrosis (Zhou et al., 2016; Honda and Oda, 2005; Fielding et al., 2014; Raby et al., 2017).

The peritoneal cavity has a normal environment that can be disturbed by bio incompatible PD solutions, which can also harm the peritoneal membrane. Traditional (Bio-incompatible) PD solutions have non-physiological features such as low pH (acidic), high lactate and glucose concentrations (hyperglycaemic) (1.5%–4.5%) (Mortier et al., 2002), and hyperosmotic dextrose solutions to achieve a sufficient UF gradient across the peritoneal membrane, and toxin elimination by maintaining electrolyte homoeostasis (Szeto and Johnson, 2017; McIntyre, 2007). However, they also trigger fibrosis, oxidative stress and microinflammation in the peritoneum, leading to changes in its structure and function which involves PMCs depletion and basement membrane breakdown leading to ultrafiltration failure, discontinuation of PD, and an increased risk of developing EPS (Terri et al., 2021; Jagirdar et al., 2019; Kang, 2020). Furthermore, heat sterilisation of PD solutions results in glucose instability generating toxic glucose degradation products (GDPs), glyoxal, 3,4-dideoxyglucosone-3-ene, methylglyoxal (MGO), and others (Jörres, 2012; Catalan et al., 2005). MGO is an extremely toxic GDPs that causes oxidative stress and peritoneal injury as well as reported to enhance the production of vascular endothelial growth factor (VEGF), which may lead to vascular permeability (Hishida et al., 2019). Furthermore, many GDPs such as 3,4-dideoxyglucosone-3-ene (3,4-DGE) are reactive carbonyl compounds which along with glucose form advanced glycation end-products (AGEs), binds to free amino groups on membrane proteins or lipids contributing to pathophysiological alterations in the peritoneal membrane (Linden et al., 2002; Mortier et al., 2004). Bio-incompatible PD solutions recruit immune cells such as Th17, gdT cells, and neutrophils to the sub-mesothelial zone, where they produce inflammatory cytokine, IL-17A.

Most important, the role of IL-17A in peritoneal membrane injury and other PD-related complications has been recently explored (Marchant et al., 2020). This cytokine activates the NF-κB pathway in PMCs, which drives the expression of factors such as IL-6 which then activates the JAK/STAT pathway promoting EMT in PMCs (Marchant et al., 2020).

During peritoneal membrane injury in PD, IL-17A production in the local site stimulates the release of more pro-inflammatory mediators, such as cytokines and chemokines, from infiltrating cells and resident peritoneal cells. This amplifies the inflammatory response promoting fibrosis and angiogenesis in the peritoneal membrane (Marchant et al., 2020).

Oxidative and cellular stress plays a key role in the peritoneal membrane damage. Free radicals in the PD effluent indicate a higher risk of technique failure in stable patients (Morinaga et al., 2012; Xu et al., 2015). Hypertonic PD solutions with high glucose and/or low pH induces oxidative stress and cause apoptosis and autophagy PMCs (Simon et al., 2017; Hara et al., 2017; Wu et al., 2018). Peritoneal membrane damage further involves mitochondrial mechanisms (Ramil-Gómez et al., 2021) pertaining to the Reactive Oxygen Species (ROS) production (López-Armada et al., 2013). In a study the authors measured mitochondrial reactive oxygen species (mtROS) and membrane potential in PMCs with different phenotypes. They found that fibroblast-like PMCs had more mtROS and less membrane potential than epithelial-like PMCs. They also demonstrated that mtROS induced EMT in omental MCs, which was prevented by mitoTEMPO. Moreover, they showed that mitochondrial DNA (mtDNA) levels in PMCs correlated positively with dialysate/plasma creatinine ratio (D/P Creat) and negatively with UF (Ramil-Gómez et al., 2021; López-Armada et al., 2013).

These results suggest that mitochondrial dysfunction drives EMT in PMCs leading to peritoneal membrane damage. Furthermore, pertaining to cellular stress and membrane damage, mitochondria release damage-associated molecular patterns (DAMPs-mtROS or mtDNA), recognized by the innate immune system, and thus triggering pro-inflammatory and pro-fibrotic responses by activating Toll-like receptors (TLRs), and purinergic receptors (Raby et al., 2018; Mills et al., 2017; Anders and Schaefer, 2014). Raby et al. conducted a study to evaluate the involvement of TLRs and DAMPs in PD solutions-induced membrane fibrosis. They exposed human uremic peritoneal leukocytes, PMCs and mouse peritoneal leukocytes to different PD solutions (bio-incompatible or more bio-compatible) for a prolonged period and measured the pro-inflammatory DAMPs, measured fibrotic responses at the mRNA/protein levels and assessed the role of TLR2/4 in the sterile peritoneal inflammation and fibrosis (Raby et al., 2018). A study has shown that Nucleotide-binding oligomerization domain-like receptor (NLR) family pyrin domain containing 3 (NLRP3) inflammasome, a member of the NLR family of intracellular sensors, also mediates sterile inflammation by regulating the release of the pro-inflammatory cytokine IL-1β which promotes peritoneal membrane damage and fibrosis (Hishida et al., 2019). Pharmacological Interventions targeting oxidative stress and their products are detailed in Table 2.

New PD solutions that are more bio-compatible have been made to mitigate the problem. Newer PD solutions, such as icodextrin, and taurin solutions, have been designed to reduce the deleterious effects of bio incompatible PD solutions exposure on the peritoneal membrane (del et al., 2016; Santamaría et al., 2015), and they help to sustain the physiological equilibrium of the peritoneal cavity. Icodextrin, is gradually absorbed from the peritoneal cavity which facilitates a sustained colloid osmotic gradient, resulting in enhanced UF compared to conventional PD solutions. Owing to its superior fluid removal capacity and reduced systemic glucose exposure, icodextrin is particularly advantageous for long-dwell PD exchanges in patients with high peritoneal solute transport rates, where it aids in optimizing volume status while minimizing the metabolic burden associated with glucose absorption (Dousdampanis et al., 2018; Ng et al., 2022). Biocompatible PD solutions, expose the patients to less Glucose Degradation Products (GDPs) than conventional solutions, and better preserve the residual renal function and diuresis with a decrease in peritonitis frequency, are created by using the following approaches: pH adjustment to neutral and GDP minimisation; bicarbonate (±lactate) buffering; glucose polymer substitution for dextrose (which also reduces pH along with GDP); and amino acids as osmotic agents (Htay et al., 2018; Zhou et al., 2016; Yohanna et al., 2015) (Table 3). Recent evidence suggests, sodium-glucose cotransporters (SGLTs) and glucose transporters (GLUTs) in the peritoneal membrane mediate glucose handling and absorption during glucose-based PD (Balzer et al., 2020).

**TABLE 3:** Therapeutic Interventions in PD solutions.

Approaches	Mechanism of action(s)	Target(s)	PD model(s)	References
New PD solutions (neutral or physiological-pH and low-GDP contents, lactate or bicarbonate as buffer)	More bio-compatibility, reduced systemic GDPs and AGEs, reduction of angiogenesis, inflammation, and fibrosis	Receptors of Glucose and Degradation Products	—	Szeto and Johnson (2017), Bartosova and Schmitt (2019), Blake (2018)
Icodextrin containing PD solution	Improved peritoneal membrane ultrafiltration failure and fewer episodes of fluid overload	Receptors of Glucose and Degradation Products	—	Goossen et al. (2020), George Kunthara, 2024
Amino Acids supplemented PD solutions (pH- 6.7 and GDPs free)	Increase in skeletal muscle Amino Acid uptake during PD	Protein metabolism	—	Kunthara and Wu (2024), Asola et al. (2008)
PD solutions containing L-Carnitine, xylitol, and low glucose	Preservation of the integrity of PMCs in peritoneal membrane, reduced fibrosis, oxidative stress, inflammation, Mitigation of metabolic disorders	Peritoneal membrane adhesion cell receptors and Endothelial cell receptors	Primary HUVECs obtained from the umbilical cords of healthy and gestational diabetic mothers, Primary human PMCs culture inserts and exposed to the PD solution only at the apical side, mimicking the condition of a PD dwell	Bonomini et al. (2016), Piccapane et al. (2020)
Novel PD solutions (Glucose load partly replaced with L-Carnitine and xylitol)	Clinical parameters of PD patients including creatinine clearance urea Kt/V, fluid status, diuresis, and total peritoneal ultrafiltration proved to be stable	Peritoneal membrane adhesion cell receptors and Endothelial cell receptors	Ten CAPD patients treated for 4 weeks with new PD solutions (phase II, prospective, open, multicenter study-(NCT04001036)	Rago et al. (2021)
Alanyl-Glutamine (Ala-Gln) supplemented glucose PD solutions pharmacological doses	Improved peritoneal membrane integrity, reduced inflammation and fibrosis, Restoration of Disturbed Cytoprotective Mechanisms to Alleviate Endothelial Cell Damage	Endothelial cell receptors	Primary HUVEC, arterioles of PD patients following PD fluids exposure, Peritoneal dialysis effluent samples from 20 stable PD patients (prospective randomized, open-label, two-period, cross-over phase I/II study)	Herzog et al. (2020), Wiesenhofer et al. (2019)
Trans-peritoneal administration of Hydrogen (H_2_) enriched dialysate	Improved peritoneal membrane integrity, reduced inflammation, fibrosis, and oxidative stress	Receptors of Glucose and Degradation Products	6 PD patients receiving neutral low-GDP dextrose solution	Ichihara et al. (2015), Terawaki et al. (2013)
Supplementation of LiCl	LiCl counteract PD solutions-induced mesothelial cell death, peritoneal membrane fibrosis, and angiogenesis. LiCl improves mesothelial cell survival in a dose-dependent manner	αB-crystallin as the mesothelial cell protein most consistently counter-regulated by LiCl. LiCl reduced VEGF release and counteracted fibrosis- and angiogenesis-associated processes	PD Patients derived PMCs, Mouse model of Chronic PD.	Herzog et al. (2021)

Abbreviations: ALK4/7, Activin Receptor like Kinase 4/7; HPMCs, Human Periotneal Mesothelial Cells; CG, chlorhexidine gluconate; MGO, methylglyoxal; PBS, phosphate buffered saline; i. p., intraperitoneal; PEG, polyethylene glycol; BMP-7, Bone Morphogenic Protein; FSP-1, Fibroblast Specific protein 1; (DKK)-1, Dickkopf-related protein; SFRP, Secreted frizzled-related proteins; GSK-3β, Glycogen Synthetase Kinase; AdTGF, β; Adenovirus expressing TGF-β; Nrf-2, Nuclear factor erythroid 2-related factor 2; PI3K, Phosphatidylinositol 3-kinase; NF-κB, Nuclear Factor-κB; PAI-1, Plasminogen Activator Inhibitor-1; RORγt, Retinoid related orphan receptor γt; TAK1, TGF-β; activated kinase 1; MSCs, Mesenchymal Stem Cells; WT1, Wilms’ tumor 1 transcription factor; GSK-3β, Glycogen Synthetase Kinase; Nrf-2, Nuclear factor erythroid 2-related factor 2; NF-κB, Nuclear Factor-κB; MCP-1, Monocyte Chemoattractant Protein-1; PGC1α, PPARγ; coactivator-1α; HIF-1α, Hypoxia-inducible factor-1α; ACE, angiotensin converting enzyme; MR, mineralocorticoid receptor; 11b-HSD2, 11b-hydroxysteroid dehydrogenase type 2; CYP11B2, Aldosterone synthase; BRG-1-Brahma related gene-1; ZO-1, Zonula Occludens-1; SAHA, suberoylanilide hydroxamic acid; 3-DZNeP, 3-deazaneplanocin A; EZH2, Histone methyltransferase enhancer of zeste homolog 2; AP-1-Activator Protein-1; PDTC, pyrrolidine dithiocarbamate; SGLT, Sodium-Dependent Glucose Transporters; MTAC, mass transfer area coefficients; 8-OHdG, 8-Hydroxydeoxyguanosine; MDA, malondialdehyde; BET, bromodomain and extra terminal domain; Cat, Catalase; Hmox1, Heme Oxygenase 1; Sod1-Superoxide Dismutase 1; MSCs, Mesenchymal Stem Cells; HSCs, Haematopoietic Stem Cells; BM, bone marrow; GFP, green fluorescent protein; LiCl, Lithoum Chloride.

PD solutions act as double edge sword for peritoneal membrane. Biocompatible solutions for PD have some advantages over conventional bio-incompatible solutions, but they also have some drawbacks (Table 4). One of the main challenges is their higher cost in some countries, which may limit their accessibility and affordability. Another issue is the lack of clear evidence on how they affect patient-level clinical outcomes, such as survival and quality of life. Therefore, their role in clinical practice needs to be further defined and evaluated.

**TABLE 4:** Bio-incompatibility and biocompatibility of PD solutions: effects on peritoneal membrane pathophysiology.

PD solutions	Bio-incompatible PD solutions	Bio-compatible PD solutions
Osmotic agents	Glucose	Glucose, Icodextrin and Amino acids
pH	∼5.5	5.5–7.3
Degraded products/Inflammatory factors	AGEs, GDPs, Reactive Oxygen Species, lactate buffer, acidic pH	Less number of degraded products (AGEs, GDPs, Reactive Oxygen Species)
Mechanisms of inflammation/fibrosis	5-HT and TGF- β1, upregulation of TIMP release, production of inflammatory cytokines (IL-4, IL-17)	Decreased osmolality, reduction if ultrafiltration (in neutral pH solution), amino acid accumulation, acidosis, uremia (in amino acid based solution)
**Pathophysiological Features**		
Peritoneum	Cytoplasmic inclusions in mesothelial cells membrane	Thin layer of mesothelial cells with no cytoplasmic inclusions
	Abnormal surface protuberances	Intact tight junctions
	Ruptured mesothelial cells membrane, defoliation from basement membrane	Thin basement membrane supports a layer of mesothelial cells
	Sub-mesothelial compact zone thickening, especially associated with inflammation (infiltration of myofibroblasts)	Sub-mesothelial zone is characterized by a low density of mesenchymal cells (dispersed fibroblasts, uncommon mast cells)
	Sub-mesothelial compact zone exhibits elevated blood vessel density, adventitial proliferation vasculopathy, and vessel calcification	Sub-mesothelial zone is characterized by low number of small arteries, arterioles, venules and capillaries
References	Chaturvedi et al. (2024), Bartosova and Schmitt (2019), Blake (2018), Goossen et al. (2020)	Santamaría et al. (2015), Dousdampanis et al. (2018); Ng et al. (2022), Yohanna et al. (2015), Szeto and Johnson (2017), Bartosova and Schmitt (2019), Blake (2018), Goossen et al. (2020), Kunthara and Wu (2024), Kunthara and Wu (2024), Asola et al. (2008), Bonomini et al. (2016), Piccapane et al. (2020), Rago et al. (2021), Herzog et al. (2020), Wiesenhofer et al. (2019), Terawaki et al. (2013), Del Peso et al. (2008)

### 4.2 Peritonitis

Peritonitis, a significant side effect, leads to PMCs damage, fibrosis, morbidity, and technical failure (Chou et al., 2022; Perl et al., 2020). Pro-fibrotic growth factors such as TGF-β1 and Fibroblast Growth Factor-2 (FGF-2) and inflammatory cytokines like IL-1β, IL-6, and others are upregulated during peritonitis (Kawaguchi et al., 2000; Virzì et al., 2022). IL-1β expression possibly relates inflammation and peritonitis intimately to the beginning and maintenance phase of the peritoneal fibrosis (Honda and Oda, 2005; Helmke et al., 2019). Likewise, IL-6 has been well connected to inflammation for functioning as solute transport increase across the PM (Wilson, 2018; Yang et al., 2020). Macrophages are reported to be the most abundant cells in PD effluent, which defines a central role in chronic inflammation (Helmke et al., 2019; Yang et al., 2020).

CX3CR1, receptor of CX3CL1 (detected on the peritoneal mesothelium), is expressed on macrophages in the peritoneal membrane (Helmke et al., 2019). A report demonstrated, dialysate exposure induces peritoneal fibrosis through CX3CR1-CX3CL1-mediated macrophage-mesothelial communication, which could be a novel therapeutic strategy for peritoneal fibrosis (Helmke et al., 2019). Based on the current data, peritonitis-induced membrane injury is associated with reduced membrane Cregs and altered complement activation products, such as C and C5b-9. The study also compared peritoneal injuries caused by fungal and *P. aeruginosa* peritonitis with those caused by Gram-positive bacterial peritonitis. Severe peritoneal membrane injuries due to fungal and *P. aeruginosa* peritonitis could result in the loss of expression of CRegs in the peritoneum, which increases deposition of complement activation products, suggesting deleterious effects in the membrane and further impaired Creg expression (Fukui et al., 2023). Zindel et al. documented, bacterial contamination activates mesothelial EGFR signalling in postsurgical PF, leading to MC-derived myofibroblasts and peritoneal adhesion (Zindel et al., 2021). Moreover, the authors in a 20-year PD cohort examined the peritoneal membrane characteristics and found that the high peritoneal transport group had a higher risk of peritonitis than the low or intermediate transport groups, even after adjusting for demographics, comorbidities, and biochemical parameters (Chou et al., 2022).

### 4.3 Peritoneal Angiogenesis

Peritoneal membrane solute transport and angiogenesis are regulated by matrix metalloproteinase 9 (MMP9) *via* β-catenin signaling. These factors impair ultrafiltration, cause chronic hypervolemia, and increase the risk of technique failure and mortality in PD patients (Padwal et al., 2017). Authors report that MMP9 mRNA expression in PMCs from peritoneal effluent of PD patients correlates with membrane solute transport properties and suggeste suggestes a role for MMP9 in peritoneal membrane injury. They proposed that MMP9 induced peritoneal angiogenesis by cleaving E-cadherin and activating b-catenin signaling, which increased VEGF expression (Padwal et al., 2017). New blood vessels form through angiogenesis, a highly complex process that depends on the balance between growth factors that stimulate or inhibit it (Zhu et al., 2021). Peritoneal angiogenesis plays a key role in developing peritoneal fibrosis (Zhu et al., 2021). Studies showed that TGF-β1 and VEGF-A mediate fibrosis in PD patients (Kinashi et al., 2018; Kariya et al., 2018). TGF-β1 stimulates VEGF-A expression, which in turn promotes angiogenesis (Kinashi et al., 2018; Kariya et al., 2018), indicating that the TGF-β1-VEGF-A pathway plays a key role in fibrosis-associated peritoneal angiogenesis (Kariya et al., 2018).

Twist, a basic helix-loop-helix DNA-binding protein, regulates E-cadherin expression and induces EMT and MMP9 expression in PMCs. The study revealed that peritoneal membrane injury increased Twist expression in the peritoneum (Margetts, 2012).

In a uremic rodent model, angiogenesis and fibrosis have been demonstrated during PD, accompanied by increased expression of angiopoietin (Ang)-2 and reduced expression of Ang receptor Tie2 (Stone et al., 2016; Yuan et al., 2009). With bio-incompatible PD solutions, AGEs and IL-6 can promote the production of VEGF, which has been detected in the effluent of long-term PD patients and involved in inflammation (Stone et al., 2016; Yuan et al., 2009). Therapeutic interventions targeting angiogenesis in the peritoneum are detailed in (Table 2).

## 5 Molecular Mechanisms and Therapeutic targets of signalling pathways involved in Peritoneal Fibrosis

**FIGURE 4:**
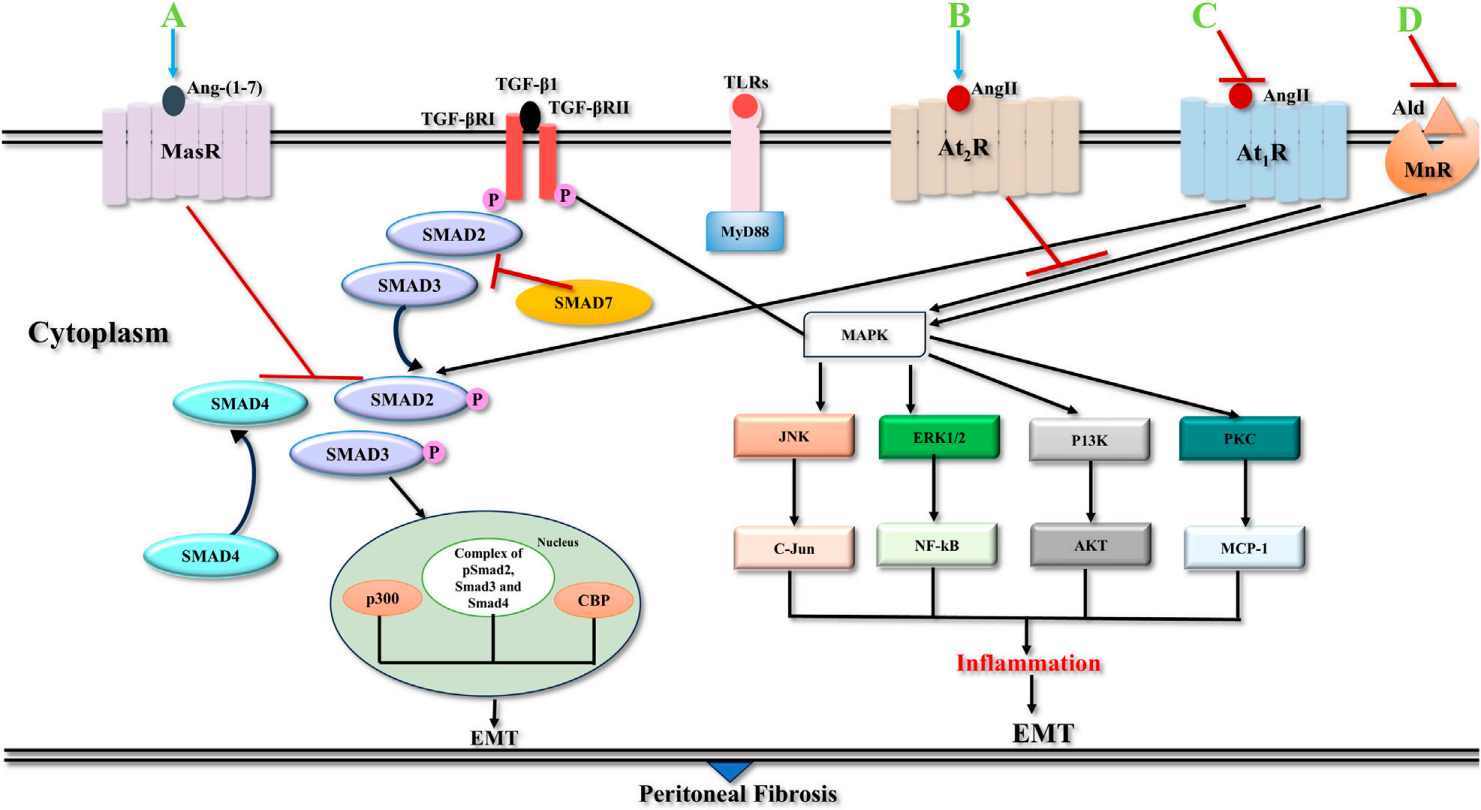
Signalling pathways in peritoneal fibrosis. TGF-βRI phosphorylates Smad2 and Smad3 and liberates them from Smad2/Smad3 receptor complex. Then after they form a Smad2/Smad3/Smad4 complex and relocate to the nuclear segment to regulate the expression of target genes with the help of various transcription factors/co-factors, CBP, p300 and others. Smad7 interferes with phosphorylation of Smad2/Smad3 by restricting their binding with TGF-β1 receptors or their movement to the nucleus. TGF-βRII and TLR ligands induce parallel pathways that culminate in the activation of various non-Smad signalling pathways, JNK, ERK1/2, PI3K, and PKC pathways which are important factors in the emergence of peritoneal fibrosis. Activation of At_1_R is involved in pro-fibrotic actions *via* classical RAAS pathway whereas At_2_R activation through alternative RAAS pathway induces anti-fibrotic actions. Binding of Ang II to At_1_R receptor activates various MAPKs, with complex of Smad2/3 and Smad4 leading to transcription of pro-fibrotic genes, i.e. *CCN2*, *COl1A1*, *PAI1*, TGF-β and PDGF. Ald activation of MnR receptor induces MAPKs and turns on the expression of pro-fibrotic proteins. Blocking At_1_R and MnR suppresses receptor activation and stop pro-fibrotic signaling. Activation of At_2_R/MasR, inhibits TGF-β fibrotic signaling. Pirfenidone might inhibit AT1R and activate MasR. Abbreviations: TGF-β1, Transforming Growth Factor Beta 1; TGF-βRI, Type I TGF-βI receptor; TGF-βRII, Type II TGF-βI receptor; CBP, Creb binding protein; TLRs, Toll like receptors; JNK, C-Jun N-terminal kinase; ERK1/2, Extracellular Signal-Regulated Kinase ½; PI3K, Phosphatidylinositol-3-kinase; PKC, Protein Kinase C; NF-kB, Nuclear Factor-kB; MCP-1, Monocyte Chemoattractant Protein-1; EMT, Epithelial to Mesenchymal transition; Ang II, Angiotensin II; Ald, Aldosterone; At_1_R, Angiotensin 1 receptor; At_2_R, Angiotensin 2 receptor; MnR, Mineralocorticoid receptor; MasR, Mas receptor; RAAS, Renin-Angiotensin- Aldosterone system; MAPKs, Mitogen activated protein kinases; *CCN2*, Cellular communication network factor 2; *COL1A1*, Alpha-1 type 1 collagen; *PAI1*, Plasminogen activator inhibitor 1; **(A)** Ang-(1–7); **(B)** Ang II; **(C)** Pirfenidone or Angiotensin convertase enzyme inhibitors (ACEIs) or Angiotensin receptor blocker (ARB); **(D)** Spironolactone.

For targeting treatments that prevent the fibrosis, it is imperative to know the molecular basis of the signalling mechanisms, which contribute to the balance between cell regeneration and activation, and maintenance of activity of myofibroblasts. Several intracellular signal transduction pathways during the process of peritoneal inflammation and angiogenesis associated with the pathophysiology behind fibrosis are depicted in Figure 4.

TGF-β1 is central in the progression of EMT concluding to peritoneal fibrosis (Gangji et al., 2009; Yao et al., 2008). TGF-β1 is a member of the growth factor family that comprises TGF-βs, activins, and (BMPs) (Xu et al., 2009). PMCs plasticity depends on TGF-β1 and BMP-7, which can induce the epithelial or mesenchymal phenotype of PMCs, respectively (Loureiro et al., 2010). Peritoneal membrane deterioration in PD patients correlates with TGF-β1 levels in PD fluids, is reported in a study (Gangji et al., 2009). Blocking peptides of TGF-β1 protect the peritoneal membrane from PD fluid-induced damage in mice (Loureiro et al., 2011). TGF-β1 signalling employs both Smad-mediated and Smad-independent mechanisms (Suryantoro et al., 2023) (Figure 4).

### 5.1 Smad TGF-β1 Signalling

The main signaling pathways responsible for the EMT process in PMCs are induced by TGF-β1 (Davies, 2004). Heterodimeric serine/threonine kinase transmembrane receptor complexes mediate the signaling of TGF-β1 factors. The ligand binds to its primary receptor (receptor type II), which recruits, trans-phosphorylates, and activates the signaling receptor (receptor type I). The receptor type I of TGF-β1, also known as activin receptor-like kinase 5 (ALK5), phosphorylates Smad2 and 3 with its serine-threonine kinase activity. BMP-7 (ALK3) receptor type I phosphorylates Smads (1, 5, and 8). The phosphorylated Smads form heterodimers with Smad4, a common mediator of all Smad pathways (Gangji et al., 2009; Xu et al., 2009; Loureiro et al., 2011). These Smad heterocomplexes move to the nucleus, binding directly to DNA and activating specific target genes. Inhibitory Smad seven limits the Smad signaling triggered by TGF-β1. They prevent the phosphorylation and/or nuclear translocation of Smad2/3 or Smad1/5/8complexes and induce their degradation by recruiting ubiquitin ligases (Gangji et al., 2009; Suryantoro et al., 2023) MMT process arises from the integration of diverse signals triggered by multiple factors, making it difficult to establish a clear hierarchy or prioritize specific pathways (López-Cabrera, 2014).

The contribution of TGF-β1-BMP7-Gremlin-1-Smad pathway cross-talk has recently been reported to be involved in peritoneal fibrosis (Ruiqi et al., 2021). The authors report a possible mechanism, Gremlin-1 enhances the TGF-β1 signalling and suppresses the expression of BMP7 and Smad1/5/8, leading to EMT and peritoneal fibrosis (Ruiqi et al., 2021). In other study, BMP-7 inhibitory effect on EMT is dependent on the activation of Smad1/5/8 proteins that counteract TGF-β1 activated Smad2/3 activity (Raby et al., 2018). Smad3 signalling is essential for TGF-*β*1 induced EMT and fibrosis, as evidenced by Smad3 knockout mice. These mice display less peritoneal fibrosis, collagen accumulation, and EMT (Patel et al., 2010). EMT and peritoneal membrane fibrotic injury are reduced by inhibitory Smad7, which blocks Smad signaling (Patel et al., 2010).

#### 5.1.1 Transcriptional Regulators

TGF-β1 activates Smad complexes that regulate the expression and activity of EMT transcription factors. Smad3 mediates the transcriptional induction of SNAIL-1 by TGF-β1 (Hoot et al., 2008). Smad3/4 also collaborates with SNAIL1 to repress the genes encoding E-cadherin and occludin in response to TGF-β1 (Vincent et al., 2009). Moreover, TGF-β1 induces ZEB1 expression, which is also modulated by non-Smad pathway (MAPK signalling), and Smad3/4 complexes interact with ZEB1 and ZEB2 to mediate TGFβ-regulated gene expression (Postigo, 2003).

Complete blocking of the Smad pathway does not entirely abolish the fibrosis, proposing that additional pathways are also convoluted in the fibrosis mechanisms (Kinashi et al., 2018).

### 5.2 Non-Smad TGF-β1 Signalling

Documentation of TGF-β1/Smads signaling help in understanding the fibrosis mechanism partially, but still, the entire phenomenon is incompletely understood. Complementing, the Smad proteins crosstalk with various non-canonical pathways to communicate downstream responses. Non-canonical pathways such as Mitogen activated protein kinases (MAPKs), Extracellular regulated Kinases (ERK1/2), and p-38, along with Jun N-terminal kinases (JNK), Src, phosphatidyl inositol 3 kinase (PI3Kinase)/AKT, Wnt/β-catenin/Ror2 signalling pathways, and NLRP3 inflammasome transduce the pro-fibrotic effects of TGF-β1 concluding to EMT and fibrosis as discussed below.

Map kinase (MEK)-ERK1/2 is a key pathway that mediates the effects of TGF-β1 on EMT and fibrosis (Gui et al., 2012). However, ERK- nuclear factor κ-light chain-enhancer of activated B cells (NF-kB)-Snail-1 pathway inhibition prevents this EMT induction. Moreover, the pathway inhibition also triggers mesenchymal to epithelial transition (MET), reverse process of EMT, in PMCs of patients on long term PD (Strippoli et al., 2008). The AMPK-NF-kB pathway mediates the anti-fibrotic molecule mechanism, which suppresses suppressing PAI-1 expression in PMCs and prevents TGF-β1 induced EMT in peritoneal fibrosis. (Strippoli et al., 2008).

In continuation of non-Smad pathways, TGF-β1 is reported to activate TAK-1 (TGF-β activated kinase 1), which mediates the phosphorylation and activation of p38 and JNK MAPKs pathway (Shim et al., 2005). The p38-mediated pathway controls the EMT process of PMCs through a feedback mechanism that reduces ERK1/2, TAK-1-NF-*κ*B activities. JNK inhibition is stated to preserve E-cadherin expression and prevents EMT conversion (Strippoli et al., 2010). JLP, a (JNK interacting protein) family scaffold protein for MAPK pathway (Dhanasekaran et al., 2007), negatively regulates peritoneal fibrosis by modulating TGF-β1/Smad signalling pathway, EMT, autophagy, and apoptosis (Tian et al., 2022).

PI3K/AKT pathway is another non-Smad mechanism that has been widely investigated in EMT process, contributes pathologically to fibrosis (Lamouille et al., 2014). A report indicated that blocking the PI3K/AKT/mTOR pathway enhanced autophagy and reduced PF during PD (Jia et al., 2022). By reducing intracellular ROS levels, this inhibition enhances the expression of ZO-1 and E-cadherin, and suppresses the expression of p-PI3K/PI3K, p-mTOR/mTOR, fibroblast-specific proteins ferroptosis suppressor protein 1 (FSP1), and α-SMA, which are associated with fibroblast differentiation, thus alleviating fibrosis (Suryantoro et al., 2023). The PI3K/AKT pathway also plays a role in EMT. Wang et al. found that AKT is overactivated during MMT (Jia et al., 2022), and the expression levels of p-AKT and α-SMA in PMCs are significantly inhibited after intervention with the PI3K/AKT pathway blocker wortmannin (Jia et al., 2022). A recent study showed that rat PMCS incorporated adipose-derived mesenchymal stem cell-derived extracellular vesicles (ADSC-EVs) and exhibited increased proliferation and migration *via* the activation of MAPK-ERK1/2 and PI3K-Akt pathways, which prevented postoperative peritoneal adhesions (Shi M. et al., 2022).

Src activation contributes to peritoneal fibrosis by stimulating the TGF-β1 pathway in a chlorhexidine-stimulated model of peritoneal membrane injury (Wang et al., 2017). Src is required for the phosphorylation of TGF-β receptor (R)-II, which activates TGF-β1. TGF-β1 plays a critical role in EMT programming *via* Smad and non-Smad RAS/RAF/MEK/ERK pathways; the PI3K/AKT/mTOR pathway; and the STAT3 pathway, which regulate the expression of SNAIL, c-Myc and Cyclin D1 (Wilson et al., 2020).

#### 5.2.1 Transcriptional Regulators

Snail, the master factor of EMT, inhibits E-cadherin expression directly. Both Smad and non-Smad pathways converge on the activation of Snaill (Cano et al., 2000). MEK-ERK1/2-Snail-1 pathway modulates E-cadherin and ZO-1 expression, which are essential for peritoneal membrane integrity. Blocking this pathway restored E-cadherin and ZO-1 levels, decreased peritoneal fibrosis, and enhanced membrane function (Strippoli et al., 2015). SNAIL expression in PMCs is negatively regulated by Smad3, MEK-ERK1/2, and NF-*κ*B signaling pathways (Patel et al., 2010; Strippoli et al., 2008). SNAIL and TWIST, transcription factors that mediate EMT, are under the control of NF-*κ*B, which works together with Snail to turn on the transcription of fibronectin (FN1) (Stanisavljevic et al., 2011; Šošić et al., 2003). Inhibition of AKT decreases the level of SNAIL-1 expression (Lamouille et al., 2012). SNAIL and p38 inhibition have similar effects on PMCs, while TWIST expression increases (Strippoli et al., 2010).

Evidence suggests that Heat Shock Proteins (HSPs) and Notch are involved in EMT other than kinase families. Notch signaling gets activated in the peritoneal membrane of animal fibrotic models through increased HES1/Jagged1 expression (Zehender et al., 2018). In one report involving rat peritoneal membrane (Zehender et al., 2018), HSP70 secured the PMCs from EMT *via* the involvement of Smad/Non-Smad pathways (Zehender et al., 2018).

### 5.3 Toll-like Receptor (TLR) ligand-mediated signalling pathways

When peritoneal injury and oxidative stress occurs in PMCs, nuclear high mobility group box 1 (HMGB1) protein, a DAMP, leaks out of the cells. This activates NF-κB, a transcription factor that regulates inflammatory responses. The recognition of HMGB1 by pattern recognition receptors (PRRs) such as Toll-like receptors (TLRs), nucleotide-binding oligomerization domain (NOD)-like receptors (NLRs), and receptors for advanced glycation end-products (RAGE), receptors for both DAMPs and pathogen-associated molecular patterns (PAMPs), mediates this activation (Wilson et al., 2020; Liu et al., 2017). DAMPS or PAMPS activate PRRs (Raby and Labéta, 2018), which trigger inflammatory and proliferative events through NF-κB, cytokine and TGF-β1 release and IRAK signaling (IRAK) (Balzer, 2020; Raby and Labéta, 2018). These events activate EMT transcription factors SNAIL, TWIST, and ZEB, repress E-cadherin expression, and induce EMT (Wilson et al., 2020).

PMCs express TLRs such as TLR1, 2 and 5 excluding TLR4, which play an important role in the membrane inflammation process (Colmont et al., 2011). Various cell types, including peritoneal leukocytes and PMCs, express TLRs-2 and 4 in particular, which are major receptors for DAMPs (Raby et al., 2017; Anders and Schaefer, 2014; Colmont et al., 2011). TLRs recognise and respond to a wide range of DAMPs, such as HMGB-1 and HSPs (Anders and Schaefer, 2014; Chen and Nuñez, 2010). TLRs trigger MyD88 dependent signalling pathway upon ligand binding, leading to the activation of ERK1/2, JNK, p38 MAPKs, and NF-kB, as well as the secretion of proinflammatory cytokines, such as IL-6, IL-1β and TNF-α (Colmont et al., 2011). IL-1β induces NF-κB response more strongly than TGF-β1 in PMCs, and their co-stimulation results in an additive effect. NF-κB inhibition prevents EMT induction by TGF-*β*1/IL-1β costimulation and partially reverses EMT in PMCs obtained from PD patients (Strippoli et al., 2008).

### 5.4 Vascular Endothelial Growth Factor (VEGF) Signalling

Vascular endothelial growth factor (VEGF) is a specific mitogen of vascular endothelial cells (Lamouille et al., 2014). VEGF belongs to a gene family that includes VEGFA, placental growth factor, VEGFB, C, and D. IL-6, IL-1β, IL-8, MCP-1, TNF-α, and Prostaglandin E2, all involved in angiogenesis, endothelial cells survival, proliferation and capillary tube formation (Colmont et al., 2011). Inhibiting VEGF expression could reduce pathological angiogenesis in the membrane of the long-term PD patients, a report documents (Colmont et al., 2011) Another study documents, inhibiting VEGF attenuates fibrosis, by regulating TGF-β1 expression through the phosphoinositide 3-kinase (PI3K)/Akt pathway (Lee et al., 2008). Peritoneal tissues of PD patients with PF express VEGF significantly more than those of PD patients without PF (Abrahams et al., 2014). Peritoneal fibrosis, involves angiogenesis of human peritoneal vascular endothelial cells (HPVECs) mediated by VEGF/VEGF receptor 2 (VEGFR2) signalling. This signalling pathway also interacts with Hippo/YAP signalling, which regulates cell proliferation and differentiation. Pharmacological inhibition of VEGF/Hippo/YAP signaling reduced peritoneal angiogenesis and prevented further damage to the peritoneal membrane (Zhu et al., 2021). High glucose activates estrogen receptor 1 (ESR1) in PMCs, which induces EMT and peritoneal fibrosis in long term PD. ESR1 transcriptionally regulates H19, a long non-coding RNA that interacts with the transcriptional coactivator p300 to enhance the expression of VEGFA. Targeting the ESR1/H19/VEGFA pathway pharmacologically can attenuate high glucose-induced peritoneal fibrosis (Zhao et al., 2023).

### 5.5 NOD-like receptor protein 3 (NLRP3)/IL-1β signalling

PM inflammation and fibrosis are associated with the activation of NLRP3 inflammasome, an intracellular complex of the innate immune system. It activates caspase-1 and controls the secretion of cytokines IL-18 and IL-1β in response to PD solutions with high glucose content (Hishida et al., 2019).

### 5.6 Wnt/β-catenin/Ror2 Signalling

The wingless-type mouse mammary tumor virus integration site family (WNT) signalling pathway has two branches: the canonical branch, which depends on β-catenin, and the non-canonical branch, which does not. The canonical WNT signalling pathway interacts with the TGF-β1 pathway and enhances fibrosis in various organs (He et al., 2009; Guo et al., 2012). Wnt/β-catenin signaling pathway is involved in the EMT of PMCs (Fan et al., 2021). WNT5A is a typical example of a non-canonical WNT protein (Mikels et al., 2009).

#### 5.6.1 Wnt/β-catenin Signalling

The role of WNT signalling in peritoneal membrane injury has been recently explored. WNT signaling and WNT1 expression in MCs are activated by peritoneal fibrosis and correlate with solute transport in PD patients. TGF-β1 enhances WNT2 and WNT4 expression and induces peritoneal fibrosis in mice. Adenovirus expressing TGF-β1 (AdTGF-β) infection increases β-catenin and other WNT signaling components in the peritoneum, indicating TGF-β1/β-catenin crosstalk in peritoneal membrane injury (Padwal et al., 2018). Wnt/β-catenin signaling pathway is upregulated in peritoneal dialysate-induced peritoneal fibrosis; the EMT process is blocked by the use of recombinant human Dickkopf-related protein 1 (Wnt/β-catenin inhibitor) (Guo et al., 2017) and herbal mixture which mitigates peritoneal membrane thickness and fibrosis (Huang et al., 2022).

#### 5.6.2 Wnt/Ror2 Signalling

The non-canonical WNT pathway is poorly understood in the context of peritoneal membrane damage. WNT5A binds to Ror2 and blocks WNT/β-catenin signalling, reducing β-catenin and pGSK3β levels. WNT5A also decreases peritoneal fibrosis and angiogenesis in mice. Ror2 regulates WNT5A-induced fibronectin and VEGF expression in human mesothelial cells. WNT5A prevents peritoneal injury *via* Ror2 and WNT/β-catenin pathways (Padwal et al., 2020).

## 6 Strategies to preserve the peritoneal membrane from fibrosis

PD solutions have a vital role in the pathophysiology of peritoneal fibrosis, as mentioned above. However, the interpretation of the above information should be carefully evaluated considering the “caveat” of a possible lack of specificity, the “pharmacological approach” is especially relevant from a translational point of view, since it is possible to hypothesize the design of pharmacological treatments designed to specifically preserve or recuperate the peritoneal membrane homeostasis in PD patients. We have detailed the importance of improving biocompatibility and efficacy of PD solutions, and pharmacological interventions/stem cell treatment strategies in the mechanisms involved during peritoneal membrane fibrosis in Table 3.

### 6.1 Effluent Biomarkers to assess peritoneal dialysis performance and peritoneal fibrosis

A fibrosis marker that can be accessed in the dialysate would help us identify PD patients with peritoneal membrane deterioration and high risk of complications (Li YC. et al., 2021).

IL-6, a chronic peritoneal inflammation marker, is the main biomarker used in PD and measured in dialysis fluid (Suryantoro et al., 2023; Li YC. et al., 2021). Bacterial clearance and infection (acute or subclinical) in PD patients can be assessed by IL-6 levels in PD effluent. Certain biomarkers present in dialysis fluid, such as IL-6, IL-8, and cancer antigen-125 (CA-125), are employed to estimate the quantity of peritoneal macrophages (PMCs) and assess the overall health of the peritoneal membrane (Masola et al., 2022). Some are emerging, like inflammatory markers (Th17 secreted IL-17, T reg cells, and M1/M2 macrophages), some are excreted by PMCs such as miRNAs, aquaporin-1 (AQP-1) (Lopes Barreto and Krediet, 2013) and also markers of cell stress/ageing/senescence, advanced oxidized protein products (AOPPs) (Corciulo et al., 2019). The European Training and Research in PD Network (EuTRiPD) network covers them all (Aufricht et al., 2017). Furthermore, one study provides evidence for the role of AQP-1 as the ultrafiltration mediator in the human peritoneal membrane (Corciulo et al., 2019). Tweaking of Th17 response and boosting the Treg response, might save the peritoneal membrane from damage as explicated in the study (Corciulo et al., 2019). Exposure to hyperglycemic dialysate induces oxidative stress-mediated cellular senescence. Therefore, dialysate advanced oxidized protein products levels may reflect PMC senescence and injury. Moreover, dialysate toxicity and inflammation followed by MMT process modulate the expression of Hsp27 and Hsp72. Dialysate levels of VEGF, MMP-2, and Plasminogen activator inhibitor-1 (PAI-1), associated with PD duration, may serve as indicators of PD-linked peritoneal fibrosis, according to prospective cohort studies (Corciulo et al., 2019). Proteomics and metabolomics could find new biomarkers in PD effluent that could signal PM problems. The metabolic state in PD effluent could tell how healthy the membrane is and how long it will survive. Metabolic profiling of serum and PD effluent may facilitate the evaluation of PM permeability and the early detection of PM dysfunction. Furthermore, metabolite-enriched PD solutions may prevent membrane inflammation and fibrosis, thereby preserving the peritoneal membrane’s permeability (Devuyst et al., 1998). One study suggested that effluent decoy receptor-2 (eDcR2) levels may reflect peritoneal fibrosis in PD patients (Kondou et al., 2022). Moreover, serum α-Klotho and galectin-3 levels were associated with peritoneal membrane thickness and PD duration (Yang et al., 2022). A study has shown that increased dialysate-to-plasma (D/P) creatinine ratio is associated with increased risk of mortality in PD patients (Padwal et al., 2018). The same study has demonstrated that the WNT1 gene and protein expression are correlated with peritoneal solute transport rate (PSTR) measured by D/P creatinine. Therefore, WNT1 may be a biomarker for clinical outcomes in PD patients (Padwal et al., 2018). Osteopontin, a phosphorylated glycoprotein involved in inflammation, EMT, and fibrosis may also be a useful indicator of peritoneal deterioration in long term PD patients (Li J. et al., 2021). Regression analysis of 109 PD patients showed Osteopontin levels in peritoneal effluents were an independent predictive factor for the increased PSTR (Li J. et al., 2021; Kothari et al., 2016) Effluent dialysate mtDNA levels could serve as a prognostic biomarker of peritoneal membrane damage induced by PD (Ramil-Gómez et al., 2021; Xie et al., 2019). High CCL8 levels in PD effluents may be associated with an increased risk of PD failure, and the CCL8 pathway is associated with PF (Lee et al., 2023). A study, using Fourier transform infrared (FTIR) spectroscopy, characterized the molecular profiles of PD effluents and clinical data, and used machine learning to assess the potential of this proof-of-principle study for low-cost, high-speed diagnosis in PD (Grunert et al., 2020). A recent study, highlighting that MMT associated biomarkers and clinical data under Machine Learning models (MAUXI software) can predict endurance and different PD technique failures, opening new avenues to individual treatments (Arriero-País et al., 2025). List of Markers are detailed in Table 5.

**TABLE 5:** List of validated and Pre-clinical targets for peritoneal fibrosis during peritoneal dialysis.

S.No.	Validated markers	Preclinical markers
1		
2	IL-6	Th17 secreted IL-17
3	IL-8	T reg cells
4	Cancer antigen-125 (CA-125)	M1/M2 macrophages
5	Dialysate Total Protein	Aquaporin-1 (AQP-1
6	Dialysate Albumin	Hsp27
7	C-Reactive Protein (CRP)	Hsp72
8		VEGF
9		TNF-α
10		MMP-2
11		Plasminogen activator inhibitor-1 (PAI-1)
12		effluent decoy receptor-2 (eDcR2)
13		Serum α-Klotho
14		Galectin-3
15		WNT1
16		Osteopontin
17		CCL8
18		Effluent dialysate mtDNA

## 7 Conclusion

Long-term PD leads to peritoneal membrane thickening and, ultimately, fibrosis. The peritoneal membrane suffers less harm from PD nowadays, as catheter complications have decreased and PD solutions have become more compatible with the body. It is important to note that the development of pharmacological interventions targeting peritoneal fibrosis in PD patients is still an active area of research, and much work remains to fully understand the molecular mechanisms in peritoneal fibrosis and membrane survival. Effective therapies to prevent this process remain to be developed. Other non-pharmacological strategies, such as optimizing dialysis prescription, use of biocompatible PD solutions, and individualized patient care, also play crucial roles in managing peritoneal fibrosis and improving patient outcomes. The success and safety of the newer interventions mentioned in this narrative review need to be further evaluated through clinical trials before they can be implemented in routine clinical practice.

## 8 Future Directions

Notably, future directions can be aimed at gathering information on single-cell transcriptomic studies and knockout models to enhance the peritoneal membrane viability and developing new ways to predict, detect or monitor how the peritoneal membrane works or gets injured. This could include: 1) More sophisticated mathematical models to support the peritoneal membrane function testing, such as detailed descriptions of icodextrin, macromolecular or trans-capillary aspects of peritoneal membrane function, 2) Peritoneal Equilibration Test (PET), which measures the solute and water transfer across the PM to semi-quantitatively, a non-invasive method, assess PD performance, that should be established in the clinics, 3) Better biomarkers need to be validated and used to create reliable models of prognosis, 4) Omics-based biomarkers in PD effluent of long term PD patients have the potential to predict or diagnose peritoneal membrane dysfunction, enabling the establishment of reliable early prognostic tools and, possibly, the discovery of novel therapeutic targets, 5) Peritoneal microbiota needs to be investigated in fibrogenesis, to elucidate the molecular mechanisms developmental pathways of peritoneal fibrosis, Testing treatments that protect the peritoneal membrane could include: 1) New therapies use antibodies or proteins to change the expression of these molecules that cause inflammation, which can help stop the PMCs from transforming into fibrous tissue. For example: Based on the current knowledge and recent data, pharmacological modulation of GLUTs may antagonize glucose absorption and improve peritoneal ultrafiltration which represents a major goal for future research in PD., 2) Oral or dialysate additives that prevent inflammation or scarring of peritoneal membrane, and 3) New dialysis solutions with adjusted pH. Other targets aimed at sustaining PMCs viability and regulating intricate interplay between the peritoneal membrane immune system and PMCs may help in delaying peritoneal fibrosis. By elucidating the cellular and molecular mechanisms of peritoneal membrane fibrosis, both the fundamental and translational research can be advanced, as it may enable the development of therapeutic interventions to prevent the damage and restore the homeostasis of the peritoneal membrane. Collectively, the above documented information may open the avenue for developing a novel therapy in regulating peritoneal fibrosis in PD.

The review has some limitations, such as possible publication bias that favours positive results and distorts the evaluation of therapeutic approaches. More extensive studies are needed to identify effective targets for PD patients, especially to protect the peritoneal membrane integrity and prevent EMT. Overcoming these limitations is essential for developing safe and efficacious therapies in diverse clinical scenarios of peritoneal fibrosis.
